# The Effect of Ketoconazole on Post-Burn Inflammation, Hypermetabolism and Clinical Outcomes

**DOI:** 10.1371/journal.pone.0035465

**Published:** 2012-05-11

**Authors:** Marc G. Jeschke, Felicia N. Williams, Celeste C. Finnerty, Noe A. Rodriguez, Gabriela A. Kulp, Arny Ferrando, William B. Norbury, Oscar E. Suman, Robert Kraft, Ludwik K. Branski, Ahmed M. Al-mousawi, David N. Herndon

**Affiliations:** 1 Shriners Hospitals for Children, University of Texas Medical Branch, Galveston, Texas, United States of America; 2 Department of Surgery, University of Texas Medical Branch, Galveston, Texas, United States of America; 3 Institute for Translational Sciences and the Sealy Center for Molecular Medicine, University of Texas Medical Branch, Galveston, Texas, United States of America; 4 University of Arkansas for Medical Sciences, Little Rock, Arkansas, United States of America; 5 Sunnybrook Health Sciences Centre, University of Toronto, Toronto, Ontario, Canada; Rutgers University, United States of America

## Abstract

**Background:**

Hypercortisolemia has been suggested as a primary hormonal mediator of whole-body catabolism following severe burn injury. Ketoconazole, an anti-fungal agent, inhibits cortisol synthesis. We, therefore, studied the effect of ketoconazole on post-burn cortisol levels and the hyper-catabolic response in a prospective randomized trial (block randomization 2∶1).

**Methodology/Principal Findings:**

Fifty-five severely burned pediatric patients with >30% total body surface area (TBSA) burns were enrolled in this trial. Patients were randomized to receive standard care plus either placebo (controls, n = 38) or ketoconazole (n = 23). Demographics, clinical data, serum hormone levels, serum cytokine expression profiles, organ function, hypermetabolism measures, muscle protein synthesis, incidence of wound infection sepsis, and body composition were obtained throughout the acute hospital course. Statistical analysis was performed using Fisher’s exact test, Student’s t-test, and parametric and non-parametric two-way repeated measures analysis of variance where applicable. Patients were similar in demographics, age, and TBSA burned. Ketoconazole effectively blocked cortisol production, as indicated by normalization of the 8-fold elevation in urine cortisol levels [F(1, 376) = 85.34, *p*<.001] with the initiation of treatment. However, there were no significant differences in the inflammatory response, acute-phase proteins, body composition, muscle protein breakdown or synthesis, or organ function between groups.

**Conclusions:**

Both groups were markedly hypermetabolic and catabolic throughout the acute hospital stay. Normalization of hypercortisolemia with ketoconazole therapy had no effect on whole-body catabolism or the post-burn inflammatory or hypermetabolic response, suggesting that hypercortisolemia does not play a central role in the post-burn hypermetabolic catabolic response.

**Trial Registration:**

ClinicalTrials.gov NCT00675714; and NCT00673309

## Introduction

The hypermetabolic response to a severe burn, defined as a burn encompassing greater than 40% of the total body surface area (TBSA), evokes a catabolic state that persists long after the initial insult [1]. This response is characterized by futile substrate cycling, increased oxygen consumption, glycogenolysis, proteolysis, and lipolysis [1]–[3]. The primary mediators of this deleterious response have been thought to be endogenous catecholamines and cortisol [1], [4]. Urine cortisol levels are elevated 7–10 fold after severe burn and remain elevated beyond the acute phase [4], [5]. Hypercortisolemia is associated with whole-body catabolism, inflammation, and immune dysfunction [4], [6]–[8]. Under normal conditions, there is a negative feedback loop in the hypothalamus-pituitary-adrenal (HPA) axis. After severe stress, the HPA axis’ role is to maintain hemodynamic stability and physiologic homeostasis by controlling the release of acute-phase proteins. In response to severe physiologic stress, corticotrophin-releasing factor and arginine vasopressin, which are synthesized in the hypothalamus, activate circulating adrenocorticotropic hormone (ACTH). ACTH induces the synthesis of cortisol from the adrenal cortex. Increased levels of cortisol activate glucocorticoid receptors, which terminate the release of ACTH [9]. After severe burn injury, the negative feedback loop between cortisol and ACTH is deranged [10]. This physiologic, metabolic disruption leads to persistent elevations in cortisol in severely burned pediatric patients.

Ketoconazole is an imidazole anti-fungal agent that has been shown to diminish steroid synthesis by blocking P450-dependent enzyme systems [11], [12]. It has been shown to be effective in reducing both stimulated and basal cortisol secretion in both normal and Cushing’s (hypercortisolemic) patients [13], [14]. Ketoconazole also reduces the incidence of acute respiratory distress syndrome (ARDS) in critically ill patients and shortens length of ICU stay [15]. Preliminary studies in adult burn patients have shown that urinary cortisol excretion is decreased after one week of ketoconazole administration [8]. Although patients do not have completely normal urinary cortisol excretion, they have reduced muscle protein turnover and improved muscle protein balance [8]. The effect of ketoconazole administration on immune function, organ function, or hormonal balance has not been fully investigated in this patient population. Moreover, to our knowledge, the effect of inhibiting excess cortisol production has not been evaluated in the pediatric burn population. We hypothesized that the administration of ketoconazole to block excess cortisol production in severely burned pediatric patients during the acute hospitalization would attenuate inflammation, hypermetabolism, and protein wasting.

## Results

### Demographics

**Figure 1:**
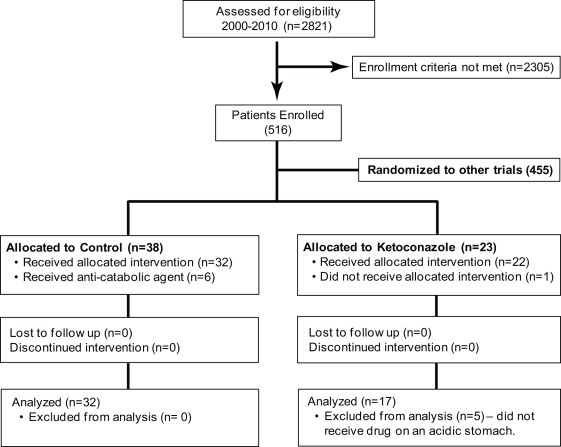
Consort Diagram.

Two-thousand eight-hundred twenty-one patients were assessed for eligibility to be enrolled in our research studies. We enrolled 516 patients (Fig. 1, Consort Diagram), 455 of whom were enrolled in studies of other anti-catabolic agents. Of the 38 patients allocated to placebo, 6 were excluded because they received anti-catabolic agents, leaving 32 patients in the standard of care/placebo group. Of the 23 patients randomized to ketoconazole, 6 were excluded. One patient did not receive the drug, while 5 were given drug under non-optimal conditions, leaving 17 patients that received standard of care plus ketoconazole treatment [per os (p.o.) on an acidic stomach] in the treatment group. There were no differences in age, gender, length of ICU stay, burn size, third-degree burn, length of ICU stay per percent burn, number of required operations, or time between operations between groups (Table 1). Incidence of inhalation injury was comparable in both groups (Table 1). Ketoconazole administration did not decrease the incidence of pneumonia, sepsis, or multi-organ failure (MOF) (Table 1).

**Table 1:** Patient demographics and the effect of ketoconazole.

	Control (n = 32)	Ketoconazole (n = 17)	*p*<.05	*p* value
Length of ketoconazole administration (days)	NA	34±8*	S	<.001
Demographics				
Age (yrs)	9±1	7±1	NS	.35
Gender (F/M)	11/21	3/14	NS	.22
XHispanic (%)	94	76	NS	.16
Caucasian (%)	6	24	NS	.16
Time to admission (days)	4±1	4±1	NS	.65
LOS ICU survivors (days)	35±4	40±7	NS	.48
TBSA burned (%)	57±3	63±6	NS	.19
3rd degree (%)	49±5	53±8	NS	.74
Flame burn (%)	92	73	NS	.97
Electrical burn (%)	4	9	NS	.97
Scald burn (%)	4	18	NS	.97
LOS/% TBSA burned survivors (days/%)	0.6±0.06	0.6±0.09	NS	.94
No. of surgeries in survivors	5±1	5±1	NS	.68
Inhalation injury (%)	13 (41%)	9 (53%)	NS	.41
Infections, Sepsis				
Number of minor infections	10 (31%)	5 (29%)	NS	1.0
Sepsis (n)	5 (16%)	3 (17%)	NS	1.0
Multi-organ failure (n)	7 (22%)	5 (29%)	NS	.72

TBSA  =  total body surface area. LOS  =  length of stay. Data are presented as means ± SEM, counts, or percentages. *Significant difference for control versus ketoconazole for corresponding parameter, *p*<.05.

**Figure 2:**
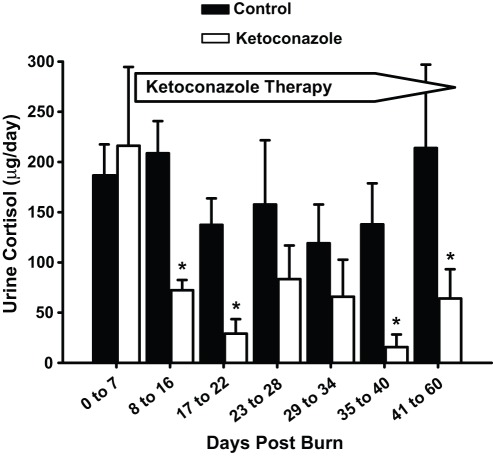
Ketoconazole administration reduces urinary cortisol levels. Data are presented as mean ± SEM. *Significant difference for control vs. ketoconazole at corresponding time point, *p*<.05. There were no significant differences between control and ketoconazole-treated patients before ketoconazole treatment. Ketoconazole therapy was initiated by the first week post burn. Urinary cortisol approached normal values in ketoconazole-treated patients and was significantly decreased during therapy.

### Cytokines, Hormones, and Proteins

**Figure 3:**
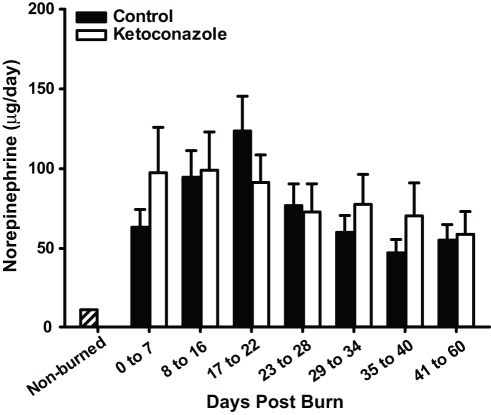
Nor-epinephrine levels are not altered by ketoconazole administration. Data are presented as mean ± SEM. Norepinephrine was significantly elevated post-burn compared to normal values (*p*<0.05).

Urinary cortisol was increased 8 fold after severe burn injury (Fig. 1). Ketoconazole treatment decreased urinary cortisol excretion to normal levels [F(1, 376) = 85.34, *p*<.001]. This effect was evident by post-burn day 8 [F(7, 376) = 8.21, *p*<.001] (Fig. 2). Catecholamine levels were significantly elevated after severe burn. Ketoconazole treatment had no effect on urine catecholamine levels [F (1, 329) = 1.08, *p* = .30] (norepinephrine shown in Fig. 3).

Confirming previous studies, we found that severe burn injury induced a vast inflammatory response. Ketoconazole did not alter any of the 17 serum cytokines measured (not shown). Serum acute-phase proteins were physiologically deranged throughout the acute hospitalization, but were not different based on treatment group. There were no differences in serum IGF-1, IGFBP-3, or rhGH between the groups. There were no differences in estrogen, free or bound testosterone, or free or bound progesterone levels between the groups. There were no significant or sustained differences in serum complement C3, α_2_-macroglobulin, haptoglobin, or C-reactive protein. Ketoconazole had no effect on triglycerides or free fatty acids.

Adrenocorticotropic hormone (ACTH or Cosyntropin) challenge tests showed no differences in responses in either patient group (Table 2). Both treatment groups were adrenally competent and responded to the challenge according to established guidelines [16].

**Table 2:** ACTH stimulation test results.

Time of measurements	Control	Mean increase from baseline	Ketoconazole	Mean increase from baseline	*p* value
Baseline	23±6	−	38±16	−	.31
30 minutes	36±6	13±3	49±24	25±9	.23
60 minutes	56±3	32±3	79±28	26±4	.26

Data are presented as means ± SEM. There were no significant differences between groups at baseline, 30 min, or 60 min and no differences in mean increases.

### Indirect Calorimetry

**Figure 4:**
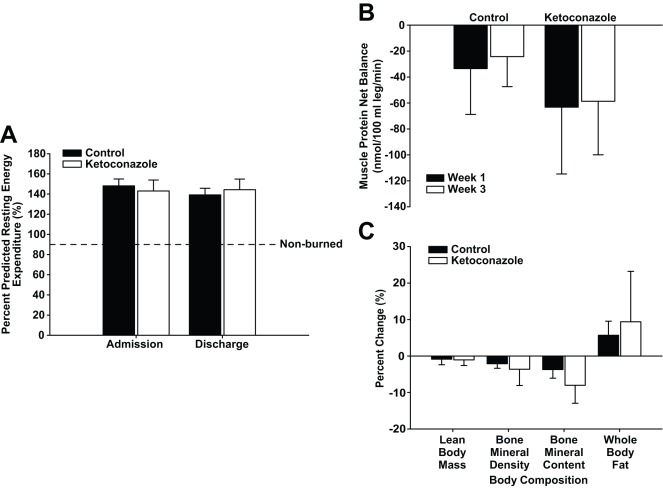
Ketoconazole administration does not alter hypermetabolism or catabolism. Data are presented as mean ± SEM. **A,** Resting energy expenditure was significantly higher in control and ketoconazole-treated patients than in non-burned volunteers. There were no significant differences between control and ketoconazole-treated patients. **B,** Changes in net protein balance induced by burn injury, or more specifically changes in muscle protein synthesis and breakdown, were measured by stable isotope studies using d5-phenyalanine infusion. Black bars indicate week one post burn and white bars week three post burn. There were no significant differences between groups. Both groups were catabolic during the study period. **C,** There was severe whole-body catabolism post burn. There were no significant differences between groups.

As previously reported, burn injury increases resting energy expenditure (REE), indicating a vast hypermetabolic response. In this study, control patients demonstrated a decrease in REE, as predicted by the Harris-Benedict equation, from 148±7% predicted at admission to 139±7% predicted at discharge (delta −9% REE) (Fig. 4a). Ketoconazole-treated patients exhibited an increase in predicted REE from 143±11% at admission (before treatment) to 144±11%, although this was not statistically significant (delta +1% REE), indicating that ketoconazole treatment had no effect on REE from admission to discharge when compared to controls.

### Muscle Protein Synthesis

Stable isotope infusion studies were used to measure muscle protein synthesis and breakdown to determine net protein balance. Burned children had a negative net protein balance in skeletal muscle at the time of the first study, at one week post admission. Peripheral muscle catabolism further increased towards the second study, which was conducted around 3–4 weeks post admission. There were no differences between control and ketoconazole for net protein balance, protein synthesis, or the fractional synthetic rate of muscle protein synthesis (Fig. 4b).

### Body Composition

Severe burn causes marked changes in body composition during acute hospitalization. Both control and ketoconazole patients experienced a 1% loss in lean body mass (LBM) during the study period. Control patients lost 2% of bone mineral density (BMD) and 4% of bone mineral content (BMC), while ketoconazole-treated patients lost 4% of their BMD and 8% of BMC (Fig. 3c). Control patients experienced 6% gains in whole-body fat, though this was not significantly different than the 9% gains in whole-body fat experienced by drug-treated patients (Fig. 3c).

### Organ Function

In the control group, liver size markedly increased after injury. Ketoconazole did not attenuate this increase in liver size compared to the control treatment (data not shown).

Predicted cardiac output (CO), cardiac index (CI), predicted heart rate (HR), predicted stroke volume (SV), and cardiac work were altered in burn patients. Ejection fraction (EF) was preserved in severely burned pediatric patients. Ketoconazole had no effect on predicted CO, CI, predicted HR, or predicted SV. Ketoconazole treatment had no effect on cardiac function (data not shown).

We further examined serum markers of organ function and homeostasis. We found that burn increased creatinine, BUN, and total bilirubin levels, while burn was associated with decreased total protein levels. Ketoconazole treatment did not have any effect on liver or renal function compared to the control treatment (data not shown).

## Discussion

Major thermal injury is followed by a profound catabolic response that persists for years after injury [1]. This catabolic state after injury leads to increased risk for infection, severe muscle wasting, morbidity, and mortality. The response has been thought to be mediated by increased plasma catecholamines, glucagon, and cortisol [4]. If left unabated, patients can have severe cardiac stress, insulin resistance, and persistent derangements in structure and function of vital organs [1], [4], [17]. It has been postulated that excess cortisol, compounded by prolonged muscular inactivity, increases skeletal muscle protein breakdown in this patient population [18]–[21]. This study was designed to reveal whether ketoconazole treatment attenuates the hypercatabolic and inflammatory response to severe burn trauma by decreasing cortisol synthesis. The principal findings in this study were that ketoconazole successfully decreased the excretion of urinary cortisol. However, it did not improve the hypermetabolic or catabolic condition of the patient population studied.

Ketoconazole blocks the 14-demethylation of lanosterol, thus preventing its conversion to cholesterol [12], [22]. It has been shown to block steroid production and cortisol production [12]. Based on the results of this study, it may be incorrect to say that ketoconazole blocks steroid production, but rather, it interferes with steroid synthesis [23]. Forty-nine patients were included in this study, with 17 receiving ketoconazole enterally at a dose of 5 mg/kg every 12 h. These 17 patients had a significant decrease in urinary cortisol excretion with ketoconazole treatment when compared to controls. Serum cortisol levels were not affected by ketoconazole treatment (data not shown). Of note, the normal circadian rhythm for serum cortisol is physiologically deranged and lost after severe burn injury, making serum cortisol an inaccurate measure of the total daily production of cortisol in our patients [24], [25]. Instead, we measured urine cortisol, a more reliable measurement of the HPA axis in our patient population [26]. There were no significant differences in the patient demographics in the two arms of the study. Adrenal function was assessed in all patients involved in the study by ACTH challenge tests. None of the patients in this study had clinical signs or symptoms of adrenal insufficiency (no response to ACTH challenge tests). Hypercortisolemia has been shown to contribute to reduced T-helper lymphocyte proliferation and immunocompetence [27]. Here, patients receiving ketoconazole did not have a lower incidence of minor infections (*p*>.05). In addition, though ketoconazole is an anti-fungal agent, there were no significant differences in the numbers of patients with sepsis or MOF.

Ketoconazole treatment did not decrease REE and thus, hypermetabolism. Both patient populations had REE that was significantly higher than normal values throughout the study period. Excess cortisol has been linked to increases in REE [5], [18]. In this study, muscle protein catabolism was elevated in control burn patients (237±57 nmol/100 ml leg/min). In the ketoconazole-treated burn patients, cortisol was reduced to normal levels, but muscle protein catabolism remained elevated (236±54 nmol/100 ml leg/min). Muscle protein synthesis is also elevated in burn patients to compensate for the increased catabolism, but the net muscle catabolism was similar with or without ketoconazole treatment (−59±41 vs.−24±23 nmol/100 ml leg/min, *p* = .57). The data suggest that the increase in muscle catabolism seen with severe burn injury is not mediated by elevated cortisol levels.

There were no significant differences in body composition between groups. Immobility confounded by hypermetabolism and the increased catabolic state led to losses in BMC and BMD. While excess cortisol has been linked to short-term bone loss [28], there were no gains in BMC or density despite ketoconazole treatment.

Severe burn injury induced a profound hyper-inflammatory response. Pro-inflammatory cytokines and acute-phase proteins were elevated throughout the study period. Ketoconazole treatment did not attenuate the inflammatory response post burn. Ketoconazole has been used to block androgen steroid synthesis in prostate cancer and been shown to cause gynecomastia in male patients in other studies [29], [30]. Ketoconazole treatment did not cause gynecomastia in these patients and did not inhibit androgen steroid synthesis, despite blocking steroid synthesis and cortisol. Ketoconazole had no effect on liver function, size, or weight compared to controls. In addition, it had no effect on cardiac function. Renal function measures did not differ between standard of care and standard of care with ketoconazole treatment.

Preliminary studies have shown an improvement in muscle protein synthesis with 7 days of ketoconazole treatment in adults [8]; however, this was not duplicated in this study, possibly due to the size of the burn studied, the pediatric population studied, or the dose of ketoconazole treatment used. Twenty-four-hour urinary cortisol in excess of 300 mcg/day is diagnostic of Cushing’s Syndrome [31]. Our patients have values that approach these levels and remain elevated long after the acute hospitalization [5]. Hypercortisolemia leads to profound muscle wasting and growth retardation [31]. Even after successful definitive pituitary surgery, patients with Cushing’s Syndrome have no significant improvement in fat mass or LBM [32]. Furthermore, urine cortisol levels return to normal levels without therapy at 3 months post burn, while muscle catabolism persists up to 9 months after injury [1], [5]. Despite reversing hypercortisolemia acutely in severe burned pediatric patients, catabolism was not reversed or attenuated. These data indicate another cause for continued muscle proteolysis.

### Conclusions

The data suggest that cortisol may not be the predominant mediator of the hypermetabolic, hypercatabolic response to severe burn injury. The effects of and elevations in catecholamines and cortisol persist well into the rehabilitative period–months to years after injury [1], [4], [17]. Significant gains in LBM, muscle protein synthesis, and multi-organ dysfunction have been achieved by blocking the effects of plasma catecholamines [17], [33]–[35]. This study attempted to isolate the potential benefits of interrupting excess cortisol in severely burned children. We have shown that attenuating cortisol levels by decreasing newly synthesized cortisol during hospitalization after the initiation of the hypermetabolic response did not diminish inflammation and hypermetabolism or alter morbidity and mortality. We conclude that efforts to abate the hypermetabolic, hypercatabolic response to stress must not exclusively address hypercortisolemia, but must inhibit the effects of catecholamines or other factors such as glucagon, either jointly or solely.

## Methods

The protocol for this trial and supporting CONSORT checklist are available as supporting information; see Checklist S1 and Protocol S1.

### Ethics Statement

This study was reviewed and approved by the Institutional Review Board of the University of Texas Medical Branch, Galveston, Texas. Prior to the study, each subject, parent, or child’s legal guardian signed a written informed consent form. All thermally-injured children had burns over 30% of their TBSA, were admitted and consented to the study protocol between 2000 and 2008, and required at least one surgical intervention. After the patient or their parent or legal guardian consented to the study, the subjects were randomized to receive ketoconazole or placebo. Ketoconazole was given enterally at a dose of 5 mg/kg every 12 h on an acidic stomach and was administered throughout the initial acute hospitalization.

### Participants

There were 38 patients randomized to control (standard of care) and 23 randomized to ketoconazole. Of the patients randomized to standard of care alone, six were excluded because they received anti-catabolic agents. Of the patients randomized to receive ketoconazole, one did not receive the drug and five patients did not receive the drug on an acidic stomach. Data from 49 severely burned patients were analyzed in this study (32 Control and 17 Ketoconazole) (Fig. 1).

Within 24 h of admission, all patients underwent total burn wound excision. Wounds were covered with 4∶1 expanded autograft or homograft [4]. After the first operative procedure, it took 5–10 days until the donor site healed and patients returned to the operating room. This approach was continued until all wound areas were covered with autologous skin material. Nutritional treatment was the same for all subjects; a caloric daily intake of 1500 kcal/m^2^ body surface +1500 kcal/m^2^ area was delivered as previously published [36]. The nutritional route of choice for our patient population was enteral, using Vivonex TEN®.

Patient demographics (age, date of burn and admission, sex, burn size, and depth of burn) and concomitant injuries such as inhalation injury, sepsis, morbidity, and mortality were recorded. Inhalation injury was diagnosed by bronchoscopy along with a consistent history. Minor infection was defined as a positive culture with less than 10^5^ colony forming units per gram of tissue or organisms. Wound infection was defined as >10^5^ colony forming units per gram of tissue in a wound biopsy with the identification of the pathogen. Repeated counts of the same bacteria in the same location were counted as the same infection. Sepsis and infection were defined by the American Burn Association and Society of Critical Care Medicine guidelines [37]–[39]. MOF was defined as previously published [4]. Wound healing was evaluated by necessary time between operative interventions–defined here by the time needed for donor sites to heal so that further autografting of the burned patient is possible. Pulmonary function was evaluated from incidence of ventilation, length of ventilation, incidence of atelectasis, and ARDS as defined by the ARDS network [40]. Pneumonia was defined by guidelines recently published by the American Burn Association [37].

### Cytokines, Hormones, and Proteins

Blood and urine were collected from each burn patient at admission, preoperatively, and 5 days postoperatively for 4 weeks and were used for analysis of serum hormone, protein, cytokine, and urine hormones. Blood was drawn in a serum-separator collection tube and centrifuged for 10 min at 1320 rpm; the serum was removed and stored at −70°C until assayed. Serum hormones and acute-phase proteins were quantified using HPLC, nephelometry (BNII, Plasma Protein Analyzer Siemens Healthcare Diagnostics, West Sacramento, CA), and ELISA techniques [4]. The Bio-Plex Human Cytokine 17-Plex panel was used with the Bio-Plex Suspension Array System (Bio-Rad, Hercules, CA) to profile expression of 17 inflammatory mediators [41].

Cosyntropin challenge tests were performed using a high performance liquid chromatography (HPLC) method on a Beckmann Coulter instrument comprising a 508 autosampler, 125 pump system, 168 DAD (diode array detector), and 24 Karate software. The column was a Symmetry Shield C18 3.5 micron, 4·5×150 mm from Waters Corporation. Mobile phase A consisted of HPLC-grade methanol with 0.1% trifluoroacetic acid (TFA). Mobile phase B was HPLC-grade water (pH = 2) with TFA. An isocratic method with 18% over 10 min was used. The analytical range is 0.1 to 10000 ng/ml. Serum samples were extracted on a HLB 1 cc 30 mg Oasis solid-phase cartridge (Waters Corporation) by conditioning with 1 ml methanol followed by 1 ml water, loading 200 µl sample in 800 µl acidified water, washing with 50% methanol in water (twice), and eluting with methanol (pH = 10.7). Eluted samples were evaporated to dry under a gentle stream of air, reconstituted in 50% methanol water (pH = 2), and subjected to HPLC analysis. Patients were fasted at least 8 h prior to the test, and measurements were taken prior to 10 a.m. A baseline blood sample was drawn, and samples were subsequently drawn at 30 min and 60 min later to determine a patient’s response.

### Hypermetabolism

#### Indirect calorimetry

All patients underwent REE measurements within one week following hospital admission and weekly thereafter during their acute hospitalization. All REE measurements were performed between midnight and 5 a.m. while the patients were asleep and receiving continuous feeding. REE was measured using a Sensor-Medics Vmax 29 metabolic cart (Yorba Linda, CA) as previously published [42]. REE was calculated from oxygen consumption and carbon dioxide production using equations described by Mlcak and colleagues [42]. Measured values were compared to predicted normal values, based upon the Harris-Benedict equation, and to body mass index [42].

#### Muscle protein synthesis

The degree of protein catabolism was quantified using stable isotope tracers. Protein kinetic studies were performed beginning between 5∶00 and 7∶00 a.m. on postoperative day five after the first excision and grafting procedure. All stable isotope studies consisted of a 5-h infusion of ^2^H_5_-Phenylalanine. Because phenylalanine is neither synthesized nor degraded in the peripheral tissues (it is metabolized only in the liver), measurement across the leg reflects the net balance of protein synthesis and breakdown. Blood samples were taken simultaneously from an ipsilateral femoral artery and vein for this determination. Indocyanine green was used to determine leg blood flow. The blood concentration of unlabeled phenylalanine was determined by gas chromatography-mass spectrometry using the internal standard approach and tert-butyldimethylsilyl esters, as previously described [43]. Indocyanine green concentrations were determined spectrophotometrically at λ = 805 nm on a Spectronic 1001 (Bausch and Lomb, Rochester, NY). As phenylalanine is neither synthesized nor degraded in the periphery, the difference in concentration of this substrate in the femoral arterial and venous plasma pools reflects the net balance of leg skeletal muscle protein synthesis and breakdown. The net balance (NB) was calculated and standardized for leg volume by the following equation: NB  =  (C*_A_* −C*_V_*) • BF, where C*_A_* and C*_V_* are free amino acid concentrations in blood from the femoral artery and vein and BF is leg blood flow in cc/min/100 ml leg. Leg blood flow was determined from a modification of Fick’s equation. BF was normalized for each patient by leg volume. Subject weight, leg circumference at prescribed points relative to anatomic landmarks, and the distances between these points were used to mathematically model leg volume [43]. Protein synthesis (PS) was calculated from the formula PS  =  (EA • CA − EV • CV) • BF/EM, where EA, EV, and EM are the amino acid enrichments in artery, vein, and muscle, respectively. Protein breakdown (PB) was calculated from the formula PB  =  PS − NB.

#### Body composition

Height and body weight were determined clinically 5 days after admission and at discharge. Total LBM, fat, BMD, and BMC were measured by dual energy x-ray absorptiometry (DEXA). A hologic model QDR-4500W DEXA (Hologic Inc, Waltham, MA) was used to measure body composition as previously published [44]–[48].

### Organ Function

M-Mode echocardiograms were used to determine CO, CI, SV, resting HR, and EF. SV and CO were adjusted for body surface area and expressed as indexes. All cardiac ultrasound measurements were obtained using a Sonosite Titan echocardiogram, with a 3.5 MHz transducer. Three measurements were performed and averaged for data analysis. Recordings were performed with subjects in a supine position and breathing freely, as recommended by the American Society of Echocardiography [4], [45]. Absolute values were then expressed as percent of normal based on published nomograms [49].

Liver ultrasound measurements were made with the HP Sonos 100 CF (Hewlett Packard Imaging Systems, Andover, MA). The liver was scanned using an Eskoline B-scanner, and liver size/volume was calculated using a previously published formula [50]. Actual size was then compared to predicted size [44]. Serum proteins, e.g., creatinine, bilirubin, and total protein were determined using standard nephelometry to evaluate organ function [4].

### Statistics

The distribution of the data was evaluated using QQ plots and the Kolmogorov-Smirnov normality test. To test for differences in normally-distributed data (cortisol, catecholamines), we conducted a two-way repeated measures ANOVA. To test for differences in non-normally distributed data (cytokines), we used two-way repeated measures ANOVA on Ranks. In either instance, we determined group differences using a *post-hoc* Bonferroni-Dunn correction to manage multiple comparisons. Two-sided equal-variance t-tests were used to compare continuous data. Fisher’s exact test was used for frequency data. *P* values less than.05 were considered significant. Continuous data are presented as mean ± SD or SEM. Frequency data are presented as counts and percentages. SAS (version 9.2) was used for data analysis and hypothesis testing. SigmaPlot (version 11.0) was used for graphics.

## Supporting Information

Checklist S1 **CONSORT Checklist.**
(DOC)

Protocol S1 **Trial Protocol.**
(DOC)
