# Supplementary material for: The Effect of Ketoconazole on Post-Burn Inflammation, Hypermetabolism and Clinical Outcomes
Source: PLoS One. 2012 May 11;7(5):e35465. doi: 10.1371/journal.pone.0035465 (PMC3350497; doi:10.1371/journal.pone.0035465)
Supplement: Protocol S1 — Trial Protocol. (DOC) [file pone.0035465.s002.doc]

**Protocol S1**

**Assessment of Mechanisms of Improved Wound Healing of Anabolic Agents, Exercise and Diet in Severely Burned Patients**

Massive burns are associated with sustained hypermetabolism characterized by elevated levels of catabolic hormones such as the catecholamine’s and cortisol, and inflammatory cytokines, such as TNF-α and IL-6. This hypermetabolic state is associated with extreme muscle wasting, delayed wound healing, and immunodeficiency. We hypothesize that during acute hospitalization and for up to two years or more post burn injury (to be followed on another protocol), administration of one or a combination of anabolic agents will moderate hypermetabolism such that lean body mass is preserved, wound healing is accelerated, and immune function is bolstered. Furthermore, we hypothesize that these improvements will be associated with decreased hospital stays and improved strength, glucagon and fat metabolism will normalize which will assist with faster reintegration into society after severe burn. Past studies have shown that all of our anabolic agents are effective in one or more of our primary physiologic outcomes, but these studies need further numbers of patients to draw more concrete conclusions and to determine underlying mechanisms. In the proposed series of studies, we intend to give one or a combination of anabolic hormones for the duration of hospitalization after a stabilization period and for up to two years or more post burn injury, and test whether our previously measured effects are still present over this duration, and secondly, whether these agents have an effect on clinically measured outcomes and mechanisms. This protocol will address issues mostly during the acute hospitalization.

# BACKGROUND AND SIGNIFICANCE

Severe injuries produce profound hypermetabolic stress responses which are characterized by increased glucose production, increased lipolysis and protein catabolism. These stress responses cause severe loss of lean body mass and muscle wasting^1-3^, delay wound healing, and compromise immunologic function^4^. All of these factors contribute to increased morbidity, mortality, and prolonged recovery from injury. The results of hypermetabolism persist for weeks, months and even years depending on the severity of the insult^2^. In the past, clinicians tried to improve the clinical status of severely injured patients during the acute hospitalization primarily with nutritional support. Administration of sufficient calories to support the hypermetabolic response to injury has been shown to reduce weight loss in severely burned patients^5^, and to decrease loss of body mass in other critically ill patients^6^. Even though weight can be maintained in severe catabolic states through nutritional support, severely injured patients still undergo massive wasting of the peripheral musculature with loss of lean body mass^7^. The catabolic response to injury leaves patients debilitated for months, and severely protracts the rehabilitation process. Therefore, investigations into treatments in addition to provision of diet to reduce injury-induced catabolism are necessary to fully overcome breakdown of lean body mass and thus improve outcomes.

Figure 1

Massive burns of >30% total body surface area (TBSA) cause severe catabolism, and are an excellent model to study the effects of injury on protein metabolism. Severe burns are characterized by dramatic increases in energy utilization which alters the metabolism of carbohydrates, fat, and protein^8^. Hormones released with the endocrine response to stress drive these metabolic changes^9-12^. As a part of the response, protein is mobilized to provide amino acids for energy as well as building blocks for host defense protein synthesis. Since no pool of stored protein exists, most of the required amino acids are taken from active muscle tissue. The increase in amino acid deamination from muscle causes muscle wasting and loss of strength, which persists despite provision of required substrate through the diet. Our group and others have been interested in mechanisms and treatment of protein catabolism after injury. We have studied and propose to continue studying the effects of hypermetabolism and catabolism on muscle protein metabolism, wound healing, and the organs that function as part of the immune system.

## Muscle Protein Metabolism after Injury

Accelerated net protein catabolism occurs after injury. When compared to normal fasted subjects^13^, protein breakdown and subsequent efflux of amino acids from muscle are elevated almost 2-fold after burn^14^. **The principal defect is an accelerated rate of protein breakdown with a failure of compensatory synthesis, resulting in a decrease in net protein synthesis (muscle protein synthesis minus muscle protein breakdown^15^**. Our previous investigation of leg muscle protein in severely burned patients confirmed decreased net protein synthesis in skeletal muscle after severe burn^16^ (Figure 1). The response does not appear to be simply from prolonged enforced bed rest^17^. In addition, we found that nutritional support with protein and amino acid intake levels several times higher than the normal requirement failed to completely reverse net protein catabolism^18^.

The catabolic response of muscle to acute stress has been studied in a number of models. The bulk of muscle protein is made up of the myofibrillar components, specifically actin and myosin. Animal studies have shown a decrease in net myofibrillar protein synthesis in response to burn^19^ and sepsis^20-23^. The contribution of myofibrillar protein constituted the majority of increased protein breakdown resulting in net protein loss^21^. In this same model, increased activity of a number of protein breakdown pathways have been shown^24^, but the most prominent by far is an increase in expression and activity of the ubiquitin-proteosome pathway^25^. Increases in gene expression of a number of protein breakdown pathways have also been shown in catabolic head-injured patients^26^.

To discuss muscle catabolism, the role of amino acid transport into and out of the muscle cell must be included. Amino acids in the cytosol from protein breakdown have two fates: transport out of the cell, or re-incorporation back into muscle protein. After burn, amino acids from protein breakdown are transported out of the muscle cell to a greater extent than they are reincorporated back into protein. Inward transport of amino acids also does not increase to compensate. The increase in breakdown and outward transport of amino acids compared to relatively decreased synthesis and inward transport result in net muscle protein loss.

Figure 2

Using our three pool model which measures protein synthesis, protein breakdown, and amino acid fluxes into and out of the cell, we have demonstrated that, in fact, *protein synthesis* ***and*** *protein breakdown are stimulated* in human muscle after severe burn^14,16^. However, increases in synthesis are outweighed by increases in breakdown. As described above, the amino acids from breakdown are directed to outward transport with related ineffective compensatory increases in synthesis.

Strategies to decrease net protein loss from muscle cells then could be directed in three fashions. The first would be to increase inward transport of amino acids (scheme 1 above). The second would be to stimulate protein synthesis which would use amino acids released from protein breakdown as the principal substrate (scheme 2). Lastly, increased protein breakdown could be inhibited (scheme 3). Inhibiting outward transport of amino acids is not physiologically possible with protein breakdown held at rates greater than protein synthesis.

By concentrating on the stimulation of protein synthesis (scheme 2), we have shown through a series of studies that muscle protein catabolism can be abated in the severely burned. A number of anabolic hormones have been used effectively to abrogate muscle catabolism in patients after severe injury when given over a portion of the hospital stay. These agents include growth hormone^27-29^, insulin-like growth factor-I^30-32^, insulin^14,16^oxandrolone^29,34,36^, testosterone, β-blockers such as propranolol, imidazoles such as itraconazole which blocks cortisol synthesis, and dehydroepiandrosterone. The great majority of these studies have been done in our laboratory. These studies have served to define the mechanisms of anabolic hormone effects on muscle protein metabolism after severe burn, and show that they indeed do work. Further questions, then, revolve around changes in clinical outcomes with anabolic agent treatment, which of the anabolic agents is best in a particular clinical situation (as some have differing mechanisms of action), and further definition of the effects of the individual anabolic agents.

This study is designed to give answers to muscle preservation looking at different agents and treatments along with changes associated with gene and protein expression. Three general classes of protein exist in the muscle: myofibrillar, sarcoplasmic, and mitochondrial. In terms of muscle function, the myofibrillar protein, specifically actin and myosin, are the most relevant, as these proteins make up the bulk of the protein mass within the cell. Investigators have shown differing kinetic responses of the three muscle protein pools in the aged^37^. However, to our knowledge, these studies have not been done in humans in response to injury or sepsis. The only current studies are those in animal models, which have shown that both myofibrillar and total net protein synthesis are decreased after burn^38^. In this proposal, we intend to measure the effects of anabolic agents *in vivo* in burned subjects on particular protein fractions, which will further define the mechanisms of action of anabolic hormones in the treatment of the severely injured. In another extension of our studies, **we propose to combine *in vivo* analysis of muscle protein kinetics at the cellular level with analysis of the molecular changes at the protein and gene level.** As a starting point to define the molecular mechanisms of insulin’s effects on muscle after severe injury, we will focus on changes in the myofibrillar protein component. Once the changes are defined at the mRNA level, and their significance is determined by the simultaneous measurement of relevant physiologic changes, we can extend these studies in later proposals to examine the causes for gene expression changes at the transcriptional and/or gene stability level. **The combination of stable isotopic measures of physiologic changes in amino acid and protein fluxes of a defined protein component and measurement of its associated molecular changes *in vivo* in critically ill human subjects will improve our knowledge of the effects of injury on muscle metabolism. Such studies have not been performed to date.**

## Wound Healing Metabolism

##

The effects of hypermetabolism on wound healing have not been well defined. Therefore, efforts to accelerate recovery from injury, of which one component is wound healing, are clearly warranted. We have previously shown that growth hormone, an agent known to increase net protein synthesis, accelerates donor site wound healing in burned patients^39,40^. These improvements were associated with increased expression of dermal-epidermal proteins, and decreased hospital stay. We have shown similar effects with insulin treatment^41^. The changes in wound healing induced by prolonged hypermetabolic states are not known, although it is suspected that the same factors causing an increase in muscle protein loss also affect cells involved in wound healing, resulting in delays in wound closure. It serves to reason, then, that stimulating anabolism in the muscle will also improve wound healing to a similar extent.

Examining skin wound protein metabolism has been difficult because of increased amino acid pool numbers and arduous measurements. For these reasons, much of the work in wound metabolism has been done *in vitro*. We developed an *in vivo* model to investigate the effects of insulin on wound healing in the burned rabbit ear^42^. This model is conducive to these studies because of its predictable blood supply, enabling accurate sampling of the amino acid kinetics in the ear in response to experimental manipulation. The rabbit ear contains only skin and cartilage; therefore detected changes are most likely related to the skin. We performed an experiment in which we measured protein kinetics in the scalded ear skin of rabbits that received one of two doses of insulin on the day of study^43^. The opposite ear which was not scalded was used as the control. We found that acute insulin infusion reduced net protein loss to near zero in the burned ear by inhibiting proteolysis at both doses. These results indicate that insulin treatment may have beneficial effects on the burn wound to accelerate healing by improving protein accretion. Unfortunately, this model cannot be applied to patients. Therefore, we are searching for other methods to explore wound protein metabolism in patients, and we continue to use more crude methods of wound healing measurement.

A significant amount of data exists to support the use of anabolic agents to improve wound healing. Overall outcomes with these treatments, however, have not been completely examined. The few studies done to determine clinical outcome measures demonstrate that treatment with growth hormone decreased length of hospital stay/% TBSA burned in severely burned children^39^, and another that documented improved mortality in burned patients treated with growth hormone^28^. The proposed studies have been designed to determine the effects of hypermetabolism in both the muscle and wounds after severe burn. In addition, we have designed the experiments to determine if these treatments are beneficial in terms of outcome, as the preliminary data suggests. Only growth hormone and insulin have been tested to date. We are now proposing to examine a number of anabolic hormones to determine their effects on wound healing using our established measures.

## Immune Organ Function

The control of the post-burn hypermetabolic response has been studied extensively in an effort to reduce the adverse effects on the burned patient. It has been shown that use of modulators, such as recombinant human growth hormone, insulin like growth factor I/IGF Binding Protein -3 (IGF-I/IGF BP-3), insulin, testosterone, oxandrolone, and itraconazole can reduce the protein catabolism associated with this condition and protect skeletal muscle from breakdown. Despite these advances, death of thermally injured patients from infectious complications remains a major concern. Severe burns are associated with immunosuppression characterized in part by an increase in type 2 T-cell cytokines (immunosuppressive) and a decrease in type 1 T-cell cytokines, and the effect of this altered protein catabolism has on immune function is not clear. Preliminary studies on small numbers of patients already receiving IGF-I/IGFBP-3 and ketoconazole have shown that treatment with anabolic agents can decrease levels of type 2 cytokines while bolstering type 1 cytokine production. In this proposal, we will also study the effects of anabolic agents on the immune system.

In order to minimize immunosuppression in burned patients, two strategies can be employed: removal of the inducer of immune suppression by excision of the burn wound^44,45^, or alternatively, the immune system could be supported to provide better protection than is provided by the compromised post-burn state.

Competent immune function is dependent on an extensive interaction of signaling between the different cell types through cytokines. Precursor T helper cells differentiate down one of two paths, Th1 and Th2 cells. Cellular immune responses are stimulated by cytokines originating from the Th1 cells, while the Th2 cells produce cytokines that stimulate the humeral immune responses. The two Th cells have a counter-regulatory effect, each able to suppress the response of the other class. Imbalances in the normal cytokine balance occur after burn, trauma, and surgical stress. The result is an increase in production of IL-4 and IL-10 as levels of IL-2 and IFN-γ are depressed. This altered cytokine environment has been shown to result in increased susceptibility to certain pathogens, notably herpes virus and *Candida albicans*.

Growth hormone (GH) has also been identified to have immune modulating properties, with receptors for growth hormone found on several classes of immune cells. Enhanced production of IFN-γ by staphylococcal enterotoxin A stimulated splenocytes occurred in the presence of GH. Immune function in burned mice infected with herpes simplex virus Type I have also been shown to be improved. Administration of insulin like growth factor-I and its binding protein, IGFBP-3, have also been shown to partially reverse the predominant Th2 response to severe burn (unpublished data).

Complete understanding of how the alterations in muscle metabolism and modulators of this process interact with the immune system has not been adequately characterized. If immune function can also be enhanced, the importance of these agents will be of even greater significance.

Ketoconazole has also been evaluated on a preliminary basis for its effects on cytokine production. Baseline Th1 cytokines, before treatment were significantly depressed relative to unburned controls (IL-2, 36±12 for severely burned vs. 501±74 for controls, and IFN-γ <40 for severely burned vs. 599±123 for controls). Th1 cytokines were greatly increased after treatment in four patients, with pre- treatment levels of IL-2 below levels of detection, and post treatment levels at 291 pg/ml, and IFN-γ levels increased from undetectable levels to 1194 pg/ml. Th2 cytokine levels were significantly elevated in the burn patients, relative to unburned controls, with IL-4 levels at 1336 pg/ml±134 vs. 176 pg/ml ± 34, and IL-10 levels 702 pg/ml ± 48 vs. 97 pg/ml ± 36. After treatment, the IL-4 levels decreased to 239.5 pg/ml (mean) and IL-10 levels decreased to 94 pg/ml. This indicates that ketoconazole is decreasing Th2 cytokines, while increasing Th1 cytokine levels. The net effect would be to promote increased cellular immunity. Steroid levels will need to be assayed to help determine the method of action of ketoconazole once a full study is undertaken.

We propose that a general conditioning program consisting of resistance and aerobic exercise will stimulate protein synthesis, bone formation, increase muscle strength and normal growth. The combination of an anabolic agent (oxandrolone or Growth Hormone) and exercise is expected to provide an additive effect on lean body mass, muscle strength, and bone mineral density.^46^ Exercise may further stimulate bone formation by increased mechanical loading^47^, or indirectly, by building up muscle strength and lean body mass.^48,49^ In addition, there is evidence that suggests that endogenous growth hormone is increased by exercise.^50^

Resistance exercise has an established influence on muscle strengthening^51^ and muscle protein synthesis.^52,53^ These beneficial effects have been well documented in both adults and adolescents. High intensity resistance training also appears to be effective in increasing muscle strength in preadolescents,^47-49^ possibly by increasing levels of neuromuscular activation and intrinsic muscle contraction and hypertrophy.^48,54^ In addition, we have previously shown that moderate resistance exercise is capable of ameliorating the decreases in skeletal muscle protein synthesis that accompany inactivity.^55^

***Resistance Exercise Training and Muscle* *Protein Metabolism***

Resistance exercise training clearly initiates hypertrophy of the trained muscles^47-49, 51-53^. Muscle hypertrophy can only result from a net positive muscle protein balance, i.e. muscle protein synthesis must be greater than breakdown. Therefore, measuring both protein synthesis and protein breakdown is important for a complete understanding of muscle anabolism and the factors that will lead to a strategy that will maximize the hypertrophic response to exercise training.

Previously, work in our laboratory demonstrated that resistance exercise improves net protein balance by increasing protein synthesis in skeletal muscle^53^. This work utilized our three-compartmental model^53^ of leg amino acid kinetics. We have subsequently confirmed this work by simultaneously assessing fractional synthetic (FSR) and breakdown (FBR) rates of skeletal muscle protein *in vivo*. The recent work of Phillips et al^56^ examined the effects of an isolated bout of resistance exercise on FSR and FBR in untrained volunteers. Subjects were studied in the fasted state on 4 occasions: at rest, and 3 h, 24 h, and 48 h after resistive exercise. Following exercise, the FBR of mixed muscle proteins was elevated at 3h and 24h. However, FBR returned to resting levels by 48h post-exercise. In contrast, the increase in FSR persisted for at least 2 days following exercise (Figure 1).

Figure 3. Fractional synthetic rate (FSR) at rest and following a resistance exercise. B. Fractional breakdown rate (FBR) at rest and following resistance exercise. Means with different letters are significantly different (P<0.05).

As a result of the elevation in FSR, the net protein balance within the exercised muscle was significantly higher at all time points post-exercise. These studies clearly indicate that resistance exercise in untrained individuals improves muscle net balance. For this reason, our conditioning program is a comprehensive program that entails resistance exercise for skeletal muscle and bone density effects, as well as aerobic exercise for cardiovascular conditioning.

**Rehabilitation of Burned Patients**

Following a thermal injury, both children and adults experience some type of long-term physical impairment with rehabilitation taking years to complete.^57^ The parameters of a successful outcome includes the return to customary and pre-injury activities.^58^ For adolescents, this means the capacity to return to school^(59.60)^  and for adults returning to work related activities. Adult patients with burns <20% of total body surface area (TBSA) require an average of 13 weeks convalescence before they are capable of returning to the work force.^57^ As the percent of TBSA burned increases, so does the amount of time required before returning to normal activities.^61^ The capability of the burned patient to resume work, return to school or reintegrate into the community is often related to a systematic rehabilitation program.^(62)^ Comprehensive rehabilitation programs, however, are not always available in burn centers throughout the United States.^63^ Most programs focus on the relief of scar contractures and do not incorporate a systematic program designed to increase musculo-skeletal strength and function. Retrospective studies indicate that up to 25% could not return to normal productivity^59, 64^ or were forced to alter their roles or job descriptions.

Loss of physical capacity, such as intolerance to standing or walking, is one primary reason for an occupational change.^64^ For the vast majority of burn patients, functional changes are overwhelmingly attributed to physical status rather than psychological or social considerations.^64^ Therefore, we feel that a successful rehabilitation program must incorporate muscle-strengthening programs. The failure to rehabilitate adequately has a direct impact on the enormous costs associated with burn injury.

Traditional inpatient rehabilitation can be expensive, costing as much as $1500/day.^61^ Our programmed care, however, is of minimal cost to the patient. It is our contention that an anabolic agent (e.g. oxandrolone, Growth Hormone, etc) and supervised exercise during rehabilitation will produce results superior to current standard rehabilitation programs. Maximum functional outcomes will be reached earlier and the expenses of an intensive, inpatient rehabilitation will be eliminated.

**RESEARCH DESIGN AND METHODS**

Subject Enrollment:

From 2004 to 2009, between Shriners Hospital for Children - Galveston (SHC-G) and the Blocker Burn Unit at the University of Texas Medical Branch (UTMB), Galveston TX approximately 900 patients per year were treated (≈ 375 children and 500 adults/yr). General demographics were as follows: 35% Caucasian, 43% Hispanic, 21% African-American, and <1% Asian Pacific or Native American. Approximately 65% were male and 35% were female. Roughly 120 patients treated in these facilities sustained burns of >30% TBSA each year, thus were eligible for enrollment. Subjects between the ages of 0 and 65 years of age with greater than 30% TBSA burns sustained within two months of admission will be enrolled for the acute hospitalization phase of the study. Subjects between the ages of 0-19 years of age with greater than 30% TBSA burns will be enrolled in the long term phase of the study. Women who are pregnant via a serum pregnancy test will be excluded prior to study.

Study Design:

(Acute hospitalization phase) After enrollment, subjects will be stratified into 3 groups by age: 0-18 years, 19-45 years, and 45-90 years, then randomized to receive either standard treatment or one or a combination of the listed anabolic agents in addition to standard treatment until discharge. At any given time, the randomization scheme will be for a total of 5 groups, as the standard treatment without anabolic agent group will be used as the control for all the treatment groups. Only 4 of the proposed anabolic agents or a combination thereof will be studied at one time at the discretion of the principal investigator. This stratification and randomization will allow for comparison of anabolic agent effects in different age groups, as we have recently found that adults are more catabolic after severe burn^65^. If no differences are found between ages, the stratified groups will be combined for analysis. It also allows for the maximum number of subjects to receive perceived benefit from anabolic agent treatment.

As soon as possible (usually within 48 hours after admission) during the ongoing burn wound resuscitation process and after randomization the anabolic agent treatment will be started. Subjects will then undergo a series of excision and grafting procedures to close the burn wounds as part of the standard treatment for severe burns. These operations will be performed about every 7-10 days over a hospitalization period that can be estimated to last 0.75-1.00 days/%TBSA burned (e.g. 45-60 days and 6 operations for a 60%TBSA burned subject). Subjects will be fed enterally with Vivonex TEN (Sandoz Nutrition Corp, Minneapolis, MN) for the duration of acute hospitalization at a calculated rate of 1500 kcal/m^2^ TBSA + 1500 kcal/m^2^ TBSA burned. These feedings will be started within 24 hours of admission. Vivonex contains 1 kcal/cc in the following caloric breakdown: 82.3% carbohydrate, 15% protein, and 4.2% fat.

Depending upon the clinical status of the research client, usually between days 3-7 after the first operation, studies will be done to assess baseline muscle kinetics and body composition. These should include, depending upon condition of patient and availability of product or machine, stable isotopic studies to assess amino acid and protein kinetics across the leg, indirect calorimetry, body composition studies consisting of dual image x-ray absorptiometry and whole body potassium counting, wound healing assessments, and immune function studies. During the stable isotopic studies, muscle biopsies will be done to assess incorporation of label into protein as well as myofibrillar protein fractional synthetic rate and gene expression.

The selected anabolic agent or combination thereof will be continued until the acute discharge, which allows for treatment over 90% of the hospitalization for a typical 60% TBSA burn. The randomized agent will continue at discharge with follow-up studies for up to two years or more. The only exception is for the acute hospitalization phase during the performance of operations for insulin, where insulin treatment will be stopped for a few hours to minimize complications associated with operative fluid shifts. Insulin will be restarted after the patient arrives back into the burn intensive care unit. At this time we do not propose to continue insulin past the acute phase as part of the randomized agents. The only exception to this would be a clinical diagnosis of insulin dependent diabetes.

Efforts will be made to maintain patients on enteral diets that our patients currently receive. Over the last 8-10 years, few patients in our burn units have required parenteral nutrition due to enteral feeding intolerance^50-52^. Changes will be made only for clinical indications. Patient safety is described in a later section. Because of the potential for side effects with some of the agents, *blinding* between some patient groups is *not possible*. When feasible, outcome variables (functional measures) will be determined by blinded observers prior to analysis.

Patients return to the operating room for repeat grafting procedures about every 7-10 days when donor sites have healed. Depending upon the clinical status of the research client, usually between days 3-7 after the second operation, another stable isotopic study with muscle biopsies will be performed to determine the effects of treatment on muscle amino acid and protein kinetics and muscle gene and protein expression between individuals *and* between groups. Indirect calorimetry will be performed to assess energy expenditure and substrate utilization. Different assessments could be performed at this time including wound healing, donor site healing and immune function measured from a blood sample. We do not expect any changes in body composition in response to treatment over this relatively short duration, therefore these studies will repeated about every month while the research client is available.

Thereafter, subjects will continue to receive staged operations until the wound is completely closed. Depending upon the clinical status of the research client, after these staged operations, a stable isotopic study including muscle biopsies and indirect calorimetry could be repeated to assess changes between POD #3-7. These additional studies may be chosen to determine if the responses to an anabolic agent treatment, that we previously showed acutely, are constant throughout the hospital course which could test our first hypothesis directly.

Once the attending burn surgeon has determined that the patient’s burns are 95% healed, the patient will be ready for discharge from the acute unit. At the time of discharge, as previously defined, we will conduct exercise evaluation of the patients.

Exercise Evaluation:

All exercise evaluations will be done in our hospital facility in Galveston. Testing personnel will be masked to the type of rehabilitation track into which that patient was randomized. The purpose of the exercise evaluation is to determine the subject’s physical functional capacity by assessing cardiopulmonary function, muscle function, muscle and bone mass in addition to joint function. Exercise evaluations as part of this IRB protocol, will be done at hospital discharge (pre-exercise). In addition, and as part of the long-term IRB protocol 04-157, exercise evaluations will be done at 12-weeks after hospital discharge (post-exercise training), and at 12 months post burn, and at 18 and 24 months post burn, and will take 4 days to conduct.

Day 1, Part 1 (morning): Cardiopulmonary Exercise Test (CPET) on a treadmill ergometer using a modified Bruce protocol. From the CPET results, we will establish the individual’s VO_2_ peak and peak heart rate. Day 1, Part 2 (afternoon): Joint function will be evaluated for knee joints using dynamic functional goniometry.

Day 2, Part 1 (morning): Assessment of Muscle Strength and Function: Although exercise training will be conducted on isotonic equipment, both isotonic and isokinetic strength will be measured to determine the patient’s ability to transfer strength. Isokinetic testing has been used in assessing back-to-work status in burned patients. Isokinetic strength will be measured using a Biodex isokinetic dynamometer. We will test right and left legs for flexion (hamstrings) and extension (quadriceps) at angular velocities of 150°/second and 180°/second using dynamometry.

Assessment of Cardiopulmonary Function

Whenever possible, we will assess gas exchange (VO_2_, VCO_2_, and V_E_) using a Modified Bruce treadmill protocol. During the progressive exercise test, gas exchange at the mouth (VO_2_, VCO_2_, and V_E_) are measured using the Medgraphics CardiO_2_ Combined O_2_/ECG Exercise System (St. Paul, MN). Data are collected breath-by-breath, measured and analyzed uses breath-by-breath analysis continuously made of inspired and expired gases, flow and volume. Oxygen uptake measurements are obtained with the children wearing a nose clip (or a mask due to mouth/nose deformities) and breathing room air through a 1-way directional valve system. Speed and angle of elevation are set with each participant walking at a speed and angle of elevation starting at 1.7 mph and 0% respectively. Thereafter, the speed and level of incline are increased every three minutes. A microcomputer automatically increases workload after an individualized walking speed and predicted values for maximal exercise capacity are entered. Subjects are constantly encouraged and the test is terminated when peak volitional effort is achieved. This test protocol allows us to obtain peak treadmill time as the primary outcome in a standardized manner, as well as gas exchange measurements even when gas exchange measurements are not possible due to facial deformities. In addition, we will obtain maximal heart rate. The criteria for VO_2_peak will include an exercise heart rate of ≥ 195 beats per minute, RER >1.05, a refusal to exercise further or an unsteady gait, and an O_2_ plateau. All cardiopulmonary tests will be performed and closely monitored according to American College of Sport Medicine guidelines. Abnormal responses to exercise (such as blood pressure drops with increased workloads) will terminate the exercise test and prompt the medical doctor in charge to evaluate the subject.

Assessment of Muscle Function:

Strength testing will be assessed using a Biodex dynamometer (Shirley, NY). The isokinetic test or muscle function test will be performed on the dominant leg extensors and tested at angular velocities of 150°/second and 180°/second to approximate a burned child’s gait. During this test, the patient is seated and the position stabilized with a restraining strap over the mid-thigh, pelvis and trunk in accordance to the Biodex Multi-Joint System 3 Testing and Rehabilitation System User’s Guide. The patient is then familiarized with the Biodex test. First, the test administrator demonstrates the procedure. Second, the test procedure is explained to the patient and third, the patient is allowed to practice the actual movement during three submaximal repetitions without load as warm-up. More repetitions are not allowed to prevent the onset of fatigue. For this test, the anatomical axis of the knee joint is aligned with the mechanical axis of the dynamometer before the test. The test administrator asks the child to perform ten maximal voluntary muscle contrac­tions (full extension and flexion). The maximal repetitions are performed consecutively without rests. Two minutes of rest are then given to minimize the effects of fatigue and the test is repeated. Values of peak torque, total work and average power are calculated by the Biodex software system. The highest peak torque, total work and average power measurements between the two trials is selected. Peak torque is corrected for gravitational moments of the lower leg and the lever arm. Following a warm-up, ten repetitions at 150°/sec are then performed, with data collected on the last eight. After a two-minute rest, the dynamometer is set to 180°/sec (reflective of faster walking speed). The child is then asked to complete ten repetitions at 180°/sec, with data collected on the last eight. Results are expressed as peak torque in Newton-meter corrected for body weight and muscle mass and average power expressed as watts and muscle total work in Joules.

Assessment of Functional Dynamic Range of Motion (knee):

Functional Range of motion (FROM) measurements will be made using the dynamometer. It is measured during the performance of leg kicks and recorded in degrees of flexion and extension.

###### FIGURE 10: OVERVIEW OF BURN PATIENT REHABILITATION PROTOCOL

**OXANDROLONE Recipients**

| **Group 1** | **Acute Hospitalization** | **Normal Convalescence** | **Home Rehab Program** | **Home Rehab Program** |
| --- | --- | --- | --- | --- |
|  |  | **Home Rehab Program** |  | |
| **Group 2** | **Acute Hospitalization** | **Normal Convalescence** | **In-Patient Rehab** | **Home Rehab Program** |
|  |  | **Home Rehab Program** |  | |

**GROWTH HORMONE RECIPIENTS**

| **Group 3** | **Acute Hospitalization** | **Normal Convalescence** | **Home Rehab Program** | **Home Rehab Program** |
| --- | --- | --- | --- | --- |
|  |  | **Home Rehab Program** |  | |
| **Group 4** | **Acute Hospitalization** | **Normal Convalescence** | **In-Patient Rehab** | **Home Rehab Program** |
|  |  | **Home Rehab Program** |  | |

**PLACEBO CONTROL**

| **Group 5** | **Acute Hospitalization** | **Normal Convalescence** | **Home Rehab Program** | **Home Rehab Program** |
| --- | --- | --- | --- | --- |
|  |  | **Home Rehab Program** |  | |
| **Group 6** | **Acute Hospitalization** | **Normal Convalescence** | **In-Patient Rehab** | **Home Rehab Program** |
|  |  | **Home Rehab Program** |  | |

ANABOLIC AGENTS, DOSES AND ADVERSE EFFECTS:

- Growth hormone: dose of 0.2 mg/kg/day given subcutaneously during acute hospitalization and 0.05-0.1 mg/kg/day long term (after discharge for up to two years post burn). Complications associated with growth hormone therapy include transient edema and arthralgias. Also of note, in a trial in Europe, growth hormone was shown to increase mortality when given to critically ill adults^67^. We have had the most experience with the use of growth hormone in critically ill children, and we did not see any differences in mortality with growth hormone utilization after sever burn, and in fact, only beneficial effects were found^68^.
- Insulin: given during acute hospitalization intravenously at a continuous basal rate of at least 0.5-1.5 μg/kg/min to maintain serum blood glucose between 80-120 mg/dL (euglycemia) as the only difference in care from the group receiving standard treatment. We have previously shown that a 3-7 day course of 2.61 mU/kg•min intravenous insulin was effective in increasing net protein synthesis in severely burned adults^14^. Our dose will be lower, and thus we expect that little additional glucose will be needed. Nonetheless, subjects will be closely monitored for clinical hypoglycemia, and supplemented with additional glucose if necessary. In the case of hyperglycemia even with insulin treatment, the insulin infusion will be increased to maintain blood glucose between 80-120mg/dl. This regimen was chosen in order to deliver the greatest amount of insulin that each patient will tolerate. If patients randomized to no insulin treatment develop clinically harmful hyperglycemia associated with glycosuria (>200 mg/dl), insulin will be administered at a dose to keep serum glucose between 150 and 200 mg/dl and prevent glycosuria until insulin treatment is no longer required. This regimen was chosen for the controls to mimic the standard of care in most intensive care units.
- Oxandrolone: dosage 0.1 mg/kg every 12 hours during acute hospitalization and long term (for up to two years or more post burn). Listed complications from oxandrolone include cholestatic jaundice, bleeding while on anticoagulants, hirsutism, and acne.
- Testosterone: dosage 200 mg IM every two weeks given during acute hospitalization only to post-pubertal males. Complications are similar to that of oxandrolone with a greater propensity toward androgenic side effects.
- Propranolol: Dosage will begin be between 0.33 and 1.0mg/kg/q6h then titrated for the desired effect of about a 25-30% reduction in the mean heart rate. Complications include bradycardia, intensification of AV block, paresthesias of the hands, thrombocytopenic purpura, mental depression, hallucinations, abdominal cramping and diarrhea, pharyngitis, agranulocytosis, and bronchospasm.
- Ketoconazole or Itraconazole: 2 mg/kg and 1 mg/kg dose respectively. Adverse reactions include anaphylaxis (extremely rare), nausea and vomiting, pruritus, and increased intracranial pressure.
- GLP, GLP-1, Byetta (Exenatide): Risks include low blood sugar or hypoglycemia.
- A combination of the above agents could also be used. For instance Insulin and Propranolol, or Oxandrolone and Propranolol could be used.

All of the above treatments are FDA approved.

At each time point noted from admission the following studies assessments should be completed as listed below:

On approximately Post Operative Day (POD) #3 - 7 (usually POD #4) after OR #1, #2, and potentially other OR’s and discharge, depending upon length of stay and the status of the patient:

Height, Weight, Vital Signs

Stable isotope infusion studies with muscle biopsies (protein kinetics and gene expression) and skin biopsies (for collagen staining)

Any surgical procedures may collect discarded tissue muscle, fat and skin for gene expression and RNA analysis, as well as muscle biomarkers (e.g. MuRF-1, MAFbx, histochemistry)

Donor and wound site healing assessments

Blood Laboratories:

CBC, Chemistry with liver function tests

Nutritional panel

Immune Function Studies (cytokines, global immune function assessments)

Hormone panel (e.g. IGF-1, IGF binding proteins, GH, insulin, thyroid hormones, osteocalcin, creatine kinase, aldolase, repulsive growth molecule C, MuRF-1, c-peptide, cortisol, glucagon, CRP, epinephrine, norepinephrine, growth hormone, prolactin, prolactin relasing hormone)

24 hour urine (e.g. catecholamine’s, free cortical, and deoxypyridinoline)

Indirect calorimetry, at rest and during exercise, Resting and Exercise Energy Expenditure

Nutritional assessments based on anthropometrics and standard biochemical analyses

Post operative days 4

Pulmonary Function Tests

DEXA (total body fat, lean body and bone mass) as soon after a*dmission as possible and discharge*

Measurement of muscle cross section area (via MRI).

Bone resorption (measured via NTx-1 in urine)

Psycho/Social assessments

#### Safety monitoring for adverse events

Post operative day 4

Body Composition Studies

Post operative day 4

Body Composition Studies

FOURTH OPERATION

FIRST OPERATION

OTHER OPERATIONS

> 30% Burn

No Treatment

Anabolic Agent

Discharge

At Discharge

Exercise Testing, DEXA, Psych Testing

Post operative days 4

Stable Isotope Studies

and Muscle Biopsies

### Patient Safety:

All study subjects will be cared for in intensive care units specifically designed for the care and monitoring of burned patients. Subjects will have continuous hemodynamic and respiratory monitoring, and frequent analysis of electrolytes and blood gases. Serum glucose will be monitored specifically on an hourly basis until a steady state of infusion of insulin is reached for those subjects receiving insulin. Thereafter, serum glucose will be monitored a minimum of 4 times a day.

Dr. Herndon will directly supervise the care of patients enrolled in this study. In his absence, either Dr. Jeschke or Dr. Lee will assume responsibility for safety monitoring of the enrolled patients. A trained intensive care nurse will attend each patient twenty-four hours a day, and a physician is present on the units at all times should he or she be required. All nursing personnel and physicians are familiar with the research treatment protocols, which are reviewed weekly at a specific meeting to discuss research on the units. Any changes in the well being of the patients will be reported immediately to Dr. Herndon or in his absence, Dr. Jeschke or Dr. Lee. Specific monitoring endpoints that require immediate intervention by the attending physician include:

- Systolic blood pressure < 90 mmHg
- Heart rate < 80% of that expected for age
- Urine output < 0.5 cc/kg/hr
- Mental confusion, seizures, lethargy, or significant change in mental status
- Development of enteral feeding intolerance
- Serum glucose < 60mg/dL
- Serum sodium > 150mEq/dL
- Cardiac arrhythmias

To ensure patient safety, the following criteria will dictate holding and possible termination from the study:

- Hypoglycemia intractable to infusion of more than 100 cc/hr of 20% dextrose solution (20 gms/hr)
- Intractable hypernatremia > 150 mEq/dL for 24 hours despite intervention
- Documented seizure activity
- Cholestatic jaundice (oxandrolone and testosterone)
- Acute asthma (propranolol)

Patients with the following pre-existing conditions will be excluded:

- Recent history of myocardial infarction (within 3 months)
- Previous diagnosis of malignancy

A Data Safety Monitoring Committee will meet regularly to monitor usage and effects and/or adverse events. This committee currently consists of Walter J. Meyer III, MD, a pediatric endocrinologist and psychiatrist and is a Professor of Pediatrics and Psychiatry; Hal Hawkins, MD, Professor, Departments of Pathology and Pediatrics, Professor, Experimental Pathology Graduate Program in the Graduate School of Biomedical Sciences, Head Pathologist, Shriners Hospital for Children, Special Shared Facility for Morphology and Pathology; Wesley S. Benjamin, RN, BSN, a research Registered Nurse who is familiar with this protocol and any adverse events. Other DSMC members will be added when available. A member of the DSMC should be present at the weekly Clinical Research meetings in which patients on this and all other protocols are reviewed. All findings will be reported to the Principal Investigator.

**Study Performance**

Stable isotopic studies examining fat kinetics, glucose kinetics, muscle and wound amino acid kinetics, protein synthesis and protein breakdown (direct incorporation techniques) will be compared in patients treated with and without anabolic agents. Results will be compared both within and between groups. These studies will be done during continuous feeding of Vivonex TEN. Studying burned patients in the fed state is appropriate as they are only fasted for surgical procedures, and it minimizes any disruption to clinical care.

As part of the standard procedures in the Shriners Burns Hospital – Galveston and the Blocker Burn Unit usually each patient has a triple-lumen subclavian line inserted for clinical care. This IV line will be used for Stable Isotope infusion, sedation and blood draws. Sedation will not interfere with the results of the study as these subjects typically receive sedatives for pain and minor procedures associated with their condition, and all groups will receive the same treatment.

Whole body glucose and lipid kinetics can be assessed using 6, 6-^2^H glucose and 1-^13^C glycerol tracers given as a constant infusion in a peripheral vein. Blood samples (20 ml total) would be taken from peripheral vein after isotopic steady state is reached (<2hrs), and analyzed for glucose and glycerol enrichment.

The protocol for measuring fractional synthesis rate (FSR) and fractional breakdown rate (FBR) involves giving a bolus of ^13^C_6_ phenylalanine, followed by a second bolus of ^15^N phenylalanine 30 minutes later. Frequent blood samples (2ml each) are taken from any available arterial or venous site (See Figure 9). Skeletal muscle biopsies are obtained from the Vastus Lateralis muscle using a 5 mm Bergstrom needle at 10 and 60 minutes following injection of the first labeled phenylalanine bolus. Punch biopsies of the donor site wound are obtained using 3 mm biopsy punch needles at 10 and 90 min following injection of the first labeled phenylalanine bolus. These procedures are done under local anesthesia (and/or conscious sedation) and yield approximately 50 mg of mixed skeletal muscle and approximately 25 mg of donor site. The samples are quickly blotted dry, frozen in liquid nitrogen, and stored at -80°C for later analysis.

Time (min)

^15^N PHE bolus

^13^C_6_ PHE bolus

0

10

20

30

40

50

60

Muscle

Blood

70

80

90

Wound

Figure 9. Protocol for measuring protein synthesis and breakdown in burned children using the tracer bolus method.

Whole body net protein synthesis and breakdown will be assessed at POD #2-4 (prior to the muscle protein kinetics protocol described above) using the single-pulse method of ^15^N- alanine and collecting the total urine output  A single oral dose of ^15^N-alanine (4mg/kg body weight) will be given to the child via Intra-Gastric (IG) tube in the evening. Urine will be collected for 24 to 36 hours after the dose, and ^15^N enrichment of urinary urea will be determined in our Metabolism Unit, using isotope ratio mass spectroscopy. Total nitrogen content of the urine will be determined using a micro-Kjeldahl apparatus. Nitrogen intake will be estimated from food intake records. Nitrogen losses will include measured urinary nitrogen and estimates of fecal and integumental losses.

In summary, this design allows for the determination of mixed muscle protein synthesis and breakdown using two independent measures. The first will be done using our direct incorporation technique which incorporates blood and intracellular enrichments of the tracers to assess intracellular synthesis and breakdown as well as amino acid transport into and out of the cells from the plasma. We will also use the whole body ^15^N alanine method to determine if the same results can be obtained without muscle biopsy tissue sampling.

Assessment of changes in myofibrillar protein synthesis and gene expression in response to anabolic treatment is a logical extension to our previous studies and proposed studies examining muscle protein and amino acid kinetics. **Determining the rate of changes in protein kinetics *and* the changes in protein component synthesis and static pool levels of specific muscle genes in the same subjects will provide a powerful tool for begin the determination of mechanisms dictating muscle wasting as well as the effects of treatment.**  To assess the hypotheses below, we will use novel methodologies on tissue samples obtained during the stable isotopic studies detailed above. We have chosen to assess protein component fractional synthetic rate and myofibrillar RNA expression as our first measurements. We are actively performing these analyses in our lab as part of other studies. The determination of myofibrillar protein synthesis in relation to the other major muscle protein components will allow us to ascertain whether decreased muscle protein breakdown after injury is specific to the myofibrillar component. Analysis of mRNA expression for these proteins will further elucidate how this response is carried out at the molecular level.

*Muscle Protein Component Fractional Synthetic Rate Analysis:*

We will assess muscle component protein synthesis by fractionating the protein from the muscle samples, and determine the component synthesis rate using enrichments from the fractionated protein pools (see methods section). This analysis will allow us to test the hypothesis that the myofibrillar protein fraction has a diminished protein synthetic rate compared to normal subjects. This analysis will also allow us to determine if the myofibrillar component of muscle protein is selectively diminished in relation to the other components, namely the sarcoplasmic and mitochondrial components.

*Gene Expression:*

Snap frozen tissue samples stored in RNA buffer will be used to isolate RNA. Total RNA per microgram of DNA will be performed followed by quantitative RT-PCR analysis for actin and myosin. Actin and myosin expression were chosen because these are the two primary components of myofibrillar protein. To our knowledge, this quantitation has not been done in subjects who are catabolic after injury. While other investigators have shown increases in expression of myosin in skeletal muscle after an overnight fast, we predict that the prolonged state of catabolism seen in severely burned subjects will be associated with decreased expression of these functional genes. The analysis of the above genes will be corroborated with the metabolic data obtained from the stable isotopic studies.

Because we have skeletal muscle RNA isolated for the above studies, we will also use these samples to perform a broad assessment of RNA expression using cDNA gene array technology. RNA from muscle will be amplified and applied to gene array membranes (Atlas Human cDNA Expression Array, CLONTECH Laboratories, Palo Alto, CA) to determine the expression of 3600 genes simultaneously. Briefly, gene-specific cDNA primers are spotted on positively charged nylon membranes, including several negative plasmid controls and housekeeping genes to serve as positive controls. Specifically, the target RNA is extracted and reverse transcribed to cDNA using the 3,600 optimized primers. Probes are then hybridized overnight. The membranes are then washed and analyzed by autoradiography and phosphoimaging. The resulting expression profiles are then compared using supplied image analysis software (Atlas Technical Manual. Protocol # PT3140-1, or something similar).

To determine changes in expression of proteins important in muscle and wound function associated with severe injury, we will compare muscle and wound biopsy samples from all burned subjects with control or anabolic treatments over the course of acute hospitalization. Muscle and donor site wound biopsies will be obtained in the fed state during stable isotope studies. We will perform similar studies to those proposed above. We would like to emphasize that these studies will be done in conjunction with our stable isotopic studies, enabling us to associate physiologic changes with any alterations in component protein synthesis and gene expression.

Improvements in amino acid kinetics in the muscle toward net protein synthesis provide the metabolic basis for improved muscle size and function. Therefore, improved net protein synthesis in response to treatment will be realized with greater lean body mass and improved muscle function. In our design, we will incorporate measures of muscle mass and function to test this notion. We will first determine changes in lean muscle mass over the hospital course and during follow-up using two complimentary methods. Our principal method will be the use of dual image x-ray absorptiometry (DEXA), a technique which measures the ratio of fat, muscle, and bone during the absorption of controlled radiation. This test is used commonly for this assessment and uses a very low dose of irradiation. The test will be performed in the first week after admission and at discharge to compare changes with and without treatment. Comparisons will be made only between groups. We currently own a DEXA imager located at the Shriner’s Hospital. This machine is used approximately 10 times a week for 20 minutes per encounter; therefore machine availability is not an issue. We routinely transport acutely burned patients to this machine for this assessment.

Within the experimental design as described above, we have the opportunity to measure wound healing rates with anabolic treatment. To address the first hypothesis, we will determine the rate of protein synthesis in the donor site, and healing wounds of enrolled patients. Healing wounds that are covered with 2:1 meshed split-thickness autograph skin will be biopsied to maintain uniformity between groups.

Briefly, wounds will be biopsied during the infusion of stable isotopes as described above. All biopsies will be done under sedation with local anesthesia. These biopsies will be blotted dry and snap frozen in liquid nitrogen until analysis. The infusion of stable isotopes to detect changes in muscle protein kinetics also allows for the measurement of protein synthesis and breakdown in the skin. With infusion of labeled phenylalanine, we will measure the rate of wound total protein synthesis by determining incorporation rates of label into protein in full thickness skin biopsies from the donor site and healing wounds. In addition, we have recently begun to develop a method of determining specific synthesis of collagen in skin by determining the incorporation of labeled alanine into the isolated collagen fraction of skin protein.

Briefly, collagen is isolated from full-thickness skin from the healing burn wound and the donor site by salt precipitation in a sodium chloride gradient column. This method has been found to isolate pure collagen. Chromatography is used to determine the peak of collagen. This sample is collected, deproteinated, derivatized, and analyzed in the gas chromatograph/mass spectrometer to determine the enrichment of alanine. Through direct incorporation methods, we can determine the rate of collagen synthesis, which may be more representative of wound healing, and as we discussed above, may be more accurate than whole tissue measurements. We chose labeled keto-isocaproic acid as the tracer because it is metabolized to alanine, which is then incorporated into collagen. The tracer chosen has a more significant change in mass to be measured, and is thus of sufficient concentration and mass to determine changes from baseline. Phenylalanine cannot be used efficiently for this purpose because it is not incorporated into collagen at any appreciable extent.

With the biopsies, we will be able to corroborate an increase in wound healing by immunohistochemical methods to quantitate expression of wound healing proteins. Tissue samples will be studied by immunofluorescent staining techniques to study the basement membrane at the epidermal-dermal junction and examined under a phase-contrast microscope. Tissues will also be evaluated by electron microscopy. Assessment by stable isotopic techniques and immunofluorescent techniques independently assess wound healing, which we propose will be altered throughout the hospital course by anabolic treatment will donor site wound healing by planimetry. We have previously used this method to determine rates of wound healing with growth hormone treatment and with insulin treatment. Comparisons will be made between groups, and within groups with time as the variable.

To test another hypothesis, donor site healing will be measured by planimetry. Briefly, the site to be studied will be a donor site harvested as part of a planned surgical procedure. The care of the donor site will not be altered from standard procedures, which at our institution consists of Scarlet Red (Sherwood Medical, St. Louis MO) impregnated fine mesh gauze. A 0.010 inch thick split-thickness skin graft will be harvested from the designated donor site and immediately covered with the dressing. Beginning on the third day following each surgical procedure, part of this study could have the site being examined, measured, and photographed using a standard camera and focal length by a trained blinded observer (Research Nurse). Using sterile technique, each corner of the gauze will be gently lifted using forceps and minimal tension to test adherence. The unattached dressing will be trimmed away. This manipulation will be done by the same observer (Research Nurse) throughout the study. The wound is considered healed with complete removal of the dressing.

Over the course of the hospitalization for study patients, the number of operations and the amount of split-thickness skin grafting will be collected. This data will be adjusted for burn wound area and compared between groups to determine the number and scope of operations required. Through these comparisons, we can determine if anabolic treatment accelerates wound healing to the extent that less surgical intervention is required. We propose that anabolic treatment would allow for faster epithelialization from skin grafts resulting in less graft loss. We also propose that anabolic treatment will promote faster re-epithelialization from second-degree wounds that will heal spontaneously. In our institution, it is common for patients to have areas of full-thickness burns that are excised and grafted combined with second degree burns in other regions of the body that are not excised at the discretion of the operating surgeon. At times, the areas that were thought to be second degree wounds do not heal satisfactorily, perhaps due to inadequate healing. These wounds are later grafted. Under this design, we will quantitate burned area (both second and third degree combined) at admission. We will then quantitate the number of operations and the area requiring skin grafts as another measure of wound healing. A treatment that accelerates wound healing would then decrease the number of operations, and decrease the area of wound that was grafted.

*Immune Function Studies:*

Blood samples (≈10 ml) may be collected prior to administration of modulators of hypermetabolism. Thereafter, when subjects are receiving an anabolic agent, another blood sample will be drawn and used to test immune function on post-operative day five during the other studies.

### *Power Analysis:*

We used lean body mass maintenance as our primary endpoint for statistical analysis. We predict a 10% improvement in change in lean body mass over the hospitalization with anabolic agent treatment. With a 15% standard deviation, this would require 20 subjects per group in each age stratification to achieve differences (60 subjects total for each anabolic agent). Therefore, to test all of the proposed agents will require ≈540 subjects. We currently enroll about 90 subjects per year into related studies, and therefore expect we can reach accrual for all the proposed studies in 5-6 years. We will of course modify these projections as new data come available, and inform the IRB should changes in subject numbers be required.

## Methods

## Model Calculations

*Fractional Synthesis Rate (FSR):*

We have developed and published several studies utilizing a model which enables the quantification of muscle amino acid transmembrane transport, as well as muscle protein synthesis and breakdown. The model provides a quantification of muscle protein synthesis. Increments of protein-bound d_5_-phenylalanine enrichment between biopsies will be calculated from gas chromatography mass spectrometry. Then FSR is calculated as follows:

FSR = [(E_p2_ - E_p1_)/(E_M_·t) · 60 · 100 (10)

where E_p1_ and E_p2_ are the enrichments of the protein-bound d_5_ -phenylalanine at the start (5 min) and end (1 hr) of the sampling period. E_M_ represents the average intracellular d_5_ -phenylalanine enrichment over the time of incorporation; and t is the time in minutes. The factors 60 and 100 are required to express FSR in percent per hour. From the FSR and measured muscle mass of the leg, a value for protein synthesis is obtained that is in the same units as the model-derived value discussed above. Thus, this approach gives us an essentially independent means to verify our conclusions drawn from the model-derived values.

*Fractional Breakdown Rate (FBR):*

One of the major limitations of previous studies of FSR is that no estimation of protein breakdown could be made simultaneously. Consequently, the traditional approach to the study of muscle protein kinetics (i.e., FSR) provided no information on the net balance between synthesis and breakdown. We have developed a new method for measuring fractional protein breakdown which is independent of the A-V model. The derivation and assumptions of the calculation for FBR are described in detail elsewhere. Briefly, however, this new method for measuring fractional protein breakdown employs a variation of the traditional precursor-product method. In this case, the product is free intracellular amino acids and the precursors are arterial blood and tissue protein. This model assumes that there is no label recycled from protein breakdown back to the free intracellular pool, thus the arterial blood is the only source of tracer in the free intracellular pool, and both arterial blood and protein breakdown provide amino acids to the free intracellular pool. This model distinguishes the rate that amino acids are released from protein from the rate that amino acids are transported from plasma to tissue by using the additional measurement of the steady state enrichments of plasma and the free intracellular amino acids.

With the systematic infusion of a labeled amino acid tracer to achieve isotopic equilibrium, the enrichment in the free intracellular pool is always lower than that in arterial blood. This is due to protein breakdown which releases amino acids that dilute the isotope enrichment in the free intracellular pool. Therefore, the enrichment difference between arterial blood and the free intracellular pool reflects the fractional contributions of amino acids from these two pools. For instance, if the arterial enrichment is 0.10 and the intracellular free enrichment is 0.05, the fractional contributions from these two sources are 50% each.

After giving the labeled phenylalanine, the decay curve of the free intracellular enrichment is decided by the arterial decay (which provides the tracer and a fraction of unlabeled amino acids) and FBR (which provides the rest of the unlabeled amino acids). Given a physiological steady state, FBR is constant and the decay curves in the arterial and intracellular pools are measurable, therefore FBR is measurable. The following is the equation used for the calculation of FBR:

$FBR=\frac{E_{F}(t_{2})-E_{F}(t_{1})}{p\int_{t_{1}}^{t_{2}} E_{A}(t)dt-(1+p)\int_{t_{1}}^{t_{2}} E_{F}(t)dt}\bullet\frac{T}{Q_{F}}$ (11)

where P = EF/(EA - EF) at isotope plateau, EA(t) and EF(t) are the arterial and intracellular enrichments, and T/QF is the ratio of bound to unbound amino acid in the tissue sample.

If one ignores the variables P and T/Q_F_ in the above equation, then the equation is simply the traditional precursor-product equation. It is necessary to introduce the variable P because the traditional precursor-product equation assumes that the product is only derived from one precursor. In this case the product is the free amino acid pool which has two sources, plasma amino acids and protein bound amino acids. Therefore, the relative contribution of these two sources to the free intracellular amino acid pool needs to be included in the calculation, which is accomplished by using the variable P. P is equal to the ratio of protein breakdown to transport of amino acids into the cell, and is calculated by determining the dilution of amino acid enrichment between plasma and the intracellular space at isotopic steady state.

The factor of T/Q_F_ is necessary to make the units of FBR comparable to FSR. The traditional precursor-product equation calculates the rate of conversion of precursor to product divided by the product pool size. In this case, this is the rate of protein breakdown divided by the free intracellular amino acid pool size. In contrast, FSR is the rate of protein synthesis divided by the bound amino acid pool size. Thus, to make the measurement of fractional protein breakdown comparable to the fractional synthesis rate, a factor T/Q_F_, the ratio of bound to free amino acid pool sizes, is applied such that the units of FBR are rate of protein breakdown divided by the bound amino acid pool size.

*Dual energy x-ray absorptiometry (DEXA):*

DEXA will be used to determine changes in whole body, leg and arm lean mass. The use of this technology for this purpose is becoming more widespread. The estimated precision of DEXA for human body composition is 2‑3%^72^. However, our recent study which investigated the precision of DEXA with 20 male and female subjects noted a coefficient of variation of 0.87% with pencil beam analysis (unpublished). Leg scans will be subdivided at the knee into upper and lower regions to allow pre to post-treatment comparisons of the tested muscle groups. Arm regions will also be separated from whole-body measures to determine treatment effects on these muscle groups. We have shown the precision (%CV) of this approach with pencil beam analysis to be 1.4%^73^.

We have previously utilized DEXA to determine the effect of 14 days of bed rest on changes in LBM. Regional analysis (Specialized Region of Interest Software, Hologic in Waltham MA) of thigh and calf areas was conducted to quantitative LBM losses in locomotory muscles. We were able to determine lean mass losses of 0.5 kg and 0.1 kg in the thighs and calves, respectively (p<0.01). Therefore, DEXA will allow the accurate determination of leg mass changes over a 2-6 month period.

*Chemical Analysis:*

All the methods required for this proposal are functioning in our laboratory and are described in our previous and present publications. Enrichments of plasma and intracellular free amino acids will be determined on the NAP-derivatives using gas chromatography mass spectrometry (GCMS). In each case, an appropriated internal standard will be added to quantify concentration. In order to determine tissue-bound d_5_-phenylalanine enrichment, we will use a modification of the technique described by Calder et al^75^. This method has the great advantage of using GCMS analysis, rather than the cumbersome technique of isolation, combustion, and IRMS analysis. Muscle samples (approximately 20 mg wet weight) are prepared and hydrolyzed with 6N HCl at 110°C for 36-48 hrs. The protein hydrolysate is then passed over a cation exchange column and dried by speed vac*.* To the dried samples, 500 µl of 3,5 HBr-propanol are added for esterification and heated at 110 °C for 60 min. Samples are then sequentially dried under nitrogen, combined with 100 µl of heptaflourobutyric anhydride, and heated at 60 °C for 20 min. To determine the enrichment of protein-bound d_5_-phenylalanine, 200 µl of the derivatized sample is then analyzed on a Finnigan MD 800 GCMS (Finnigan, San Jose, CA). Protein bound d_5_-phenylalanine enrichment is determined using chemical impact ionization with methane gas and by monitoring *m/z* 407 and 409. These ions are the m+3 and m+5 enrichments, respectively, where m+0 is the lowest molecular weight of the ion. The ratio of m+5/m+3 is used as it much more sensitive than using the traditional m+5/m+0 (used for plasma samples). Enrichment from the protein bound samples are determined using a linear standard curve of known m+5/m+3 ratios and corrected back to the absolute change in m+5 enrichment over the incorporation period. The coefficient of variation of replicate measures of the m+5/m+3 ratio for samples and standards is < 1%.

All necessary analyses are routinely performed in our laboratories. Stable isotopic enrichment of plasma free amino acids will be performed using N-acetyl N-propyl (NAP) derivatives passed through a gas chromatography mass spectrometer with chemical ionization. Selected ions at appropriate mass/charge ratios are used to calculate enrichment. Concentrations will be measured by adding an internal standard of the amino acid of interest with a different label. The enrichment of d_5_ phenylalanine will be made with HFB derivatives. Alanine enrichments in the skin will be made with HFB derivatives.

Separation of Protein Subfractions are adapted from Welle et al. whereby myofibrillar and sarcoplasmic/mitochondrial proteins are separated first by low salt buffer and centrifugation. The myofibrillar proteins are again treated with low salt buffer, homogenized, and centrifuged. The sarcoplasmic and mitochondrial proteins are further separated by centrifugation (mitochondrial fraction forms a pellet), and then the mitochondrial fraction is purified by high ion strength buffer and centrifuged. Sarcoplasmic protein is extracted with 100% ethanol and centrifugation. Myofibrillar and mitochondrial proteins are separated with 100% ethanol and centrifugation, then subsequent washes with ddH_2_O. Each protein is then dissolved in 1N NaOH and then hydrolyzed in 6N HCl. The hydrolysates are then run through cation exchange columns and dried prior to GCMS analysis.

*RNA isolation:*

Total cellular RNA is isolated from skeletal muscle using the Chomczynski RNAzol B method (Tel-Test, Friendswood, TX) with 2mL RNAzol B per 100 mg muscle. *α-actin riboprobe****:*** Dr. Laurence Kedes (University of Southern California) provides the cDNA for human α-actin. This cDNA is legated into pBSM13 for generation of riboprobes. Probes are labeled as and RPA is performed with the amount of RNA subjected to hybridization reduced to 2 μg. *β-myosin riboprobe:* Human β-myosin cDNA is obtained from the American Type Culture Collection. The β-myosin cDNA was also inserted into pBSM13 and the probes generated and assays performed as described for α-actin.

*Preparation of human peripheral blood cells:*

Peripheral blood mononuclear cells (PBMC) will be isolated from heparinized whole blood by Ficoll-Hypaque density gradient centrifugation. Neutrophils will be isolated from the erythrocyte pellet**.** Red blood cells in neutrophil preparations will be eliminated by exposure to hypotonic solution and dextran sedimentation (65-67). B cells, whole T cells, CD4+ T cells, CD4+ CD45RA+ T cells, CD4+ CD45RO+ T cells or CD8+ T cells will be purified from PBMC using cell separation columns (R&D Systems) (66-67). This process will make more than 96% pure T cell and B cell preparations. Macrophages will be purified from PBMC by fibronectin-coated Petri dishes. The process will be to take 1 x 10^7 PBMC/ml in 2 ml of RPMI 1640 medium which will be incubated for 15 min. at 37 C. At the end of the cultivation, the dishes will be washed twice with warm RPMI 1640 medium (37 C) to remove nonadherent cells from the fibronectin-coated Petri dishes. After keeping the dishes at 4 C for 60 min., the cells adherent to the plastic will be recovered by scraping with a rubber policeman. Greater than 92% purities of macrophages are obtained by this technique. NK cells will be purified from PBMC by positive selection using magnetic beads coated with anti-CD16 and anti-CD56 mAbs (Dynal, Great Neck, NY) (68). Positively selected cells will be cultured overnight at 37 C to detach them from the beads, incubated for one hour at 4 C and depleted of CD3+ cells using immunomagnetic beads (Dynal) (68). The purity of these resulting populations is greater than 95% NK cells (49). All of the purified cells will be re-suspended in RPMI 1640 medium supplemented with 10% fetal bovine serum, 2 mM L-glutamine, antibiotics and 5 x 10^-5 M 2-mercaptoethanol.

*Production and assay of chemokines:*

Chemokines assays will be prepared from PBMC *in vitro*, using 2x10^6 cells/ml PBMC which will be stimulated with anti-CD3mAb (2.5 μg/ml) for 72 hrs at 37 C. Culture fluids are harvested and assayed for human α-chemokines IL-8, MIG, and IP-10 using ELISA by manufacturer’s protocols. β‑chemokines RANTES, MCP-1, MIP-1α , and MIP-1β are prepared in the same fashion and assayed using their own specific ELISA kit.

*Production and assay of cytokines:*

Assays for cytokines will be made on PBMC preparations (2x10^6 cells/ml), which are stimulated with anti-CD3 mAb (2.5 μg/ml) and cultured for 72 hours at 37 C. Manufacturer’s protocols are followed for ELISA assay of culture fluid to quantify Th 1 cytokines IL-2 and IFN-γ, and Th 2 cytokines IL-4 and IL-10.

References:

1. Newsome TW, Mason AD, Pruitt BA. Weight loss following thermal injury. Ann Surg 178, 215-217. 1973.

2. Hart D.W., Wolf S.E., Mlcak R., Chinkes D.L., Ramzy P.I., Obeng M.K., Ferrando A.A., Wolfe R.R., Herndon D.N. Persistence of muscle catabolism after severe burn. (in press). Surgery 2000.

3. Hart DW, Wolf SE, Mlcak R, Obeng M, Lal SO, Herndon DN. Persistence of muscle catabolism after severe burn. Surgery . 2000.

4. Matsuo R, Herndon DN, Kobayashi M, Pollard RB, Suzuki F. CD4- CD8- TCR alpha/beta+ suppressor T cells demonstrated in mice 1 day after thermal injury. J Trauma 42, 635-640. 1997.

5. Gore DC, Rutan RL, Hildreth MA, Desai MH, Herndon DN. Comparison of resting energy expenditures and caloric intake in children with severe burns. J Burn Care Rehabil 11, 400-404. 1990.

6. Bower RH. Nutrition during critical illness and sepsis. New Horizons 1, 348-352. 1993.

7. Streat SJ, Beddoe AH, Hill GL. Aggressive nutritional support dies not prevent protein loss despite fat gain in septic intensive care patients. J Trauma 27, 262-266. 1987.

8. Wolf SE, Barrow RE, Herndon DN. Growth hormone and IGF-1 therapy in the hypercatabolic patient. Bailliere's Clin Endo Metab 10, 447-463. 1996.

9. Bessey PQ, Watters JM, Aoki TT, Wilmore DW. Combined hormonal infusions stimulate the metabolic response to injury. Ann Surg 200, 264-280. 1984.

10. Watters J.M., Bessey P.Q., Dinarello C.A., Wolff S.M., Wilmore D.W. Both inflammatory mediators and endocrine mediators stimulate the host response to sepsis. Arch Surg 1986; 121:179-190.

11. Gore DC, Jahoor F, Wolfe RR, Herndon DN. Acute response of human muscle protein to catabolic hormones. Ann Surg 218, 679-684. 1993.

12. Bessey P.Q., Watters J.M., Aoki T.T., Wilmore D.W. Combined hormonal infusions stimulate the metabolic response to injury. Ann Surg 1984; 200:264-280.

13. Ferrando AA, Tipton KD, Doyle D, Phillips SM, Cortiella J, Wolfe RR. Net protein synthesis and amino acid uptake with testosterone injection. Am J Physiol 275, E864-E871. 1998.

14. Ferrando AA, Chinkes DL, Wolf SE, Matin S, Herndon DN, Wolfe RR. A submaximal dose of insulin promotes net skeletal muscle protein synthesis in patients with severe burns. Ann Surg 229, 11-18. 1999.

15. Jahoor F, Desai MH, Herndon DN, Wolfe RR. Dynamics of protein metabolic response to burn injury. Metabolism 37, 330-337. 1988.

16. Sakuri Y, Aarsland AA, Chinkes DL, Nguyen TT, Pierre EJ, Wolfe RR, Herndon DN. Anabolic effects of insulin in burned patients. Ann Surg 222, 283-297. 1996.

17. Shangraw R.E., Stuart C.A., Prince M.J., Peters E.J., Wolfe R.R. Insulin responsiveness of protein metabolism *in vivo* following bedrest in humans. Am J Physiol 1988;E548-E558.

18. Shaw JHF, Wolfe RR. An integrated analysis of glucose, fat, and protein metabolism in severely traumatized patients: studies in the basal state and the response to total parenteral nutrition. Ann Surg 209, 63-72. 1989.

19. Fang CH, James JH, Ogle CK, Fischer JE, Hasselgren PO. Influence of burn injury on protein metabolism in different types of skeletal muscle and the role of glucocorticoids. J Am Coll Surg 180, 33-40. 1995.

20. Hasselgren PO, Talamini M, James JH, Fischer JE. Protein metabolism in different types of skeletal muscle during early and late sepsis in rats. Arch Surg 121, 918-924. 1986.

21. Hasselgren PO, James JH, Benson DW, Hall-Angeras M, Angeras U, Fischer JE, Li S, Fischer JE. Total and myofibrillar protein breakdown in different types of skeletal muscle: effects of sepsis and regulation by insulin. Metabolism 38, 634-641. 1989.

22. Tiao G., Fagan J.M., Samuels N., James J.H., Hudson K., Lieberman M., Fischer J.E., Hasselgren P.O. Sepsis stimulates non-lysosomal, energy dependent proteolysis and increases ubiquitin mRNA levels in rat skeletal muscle. J Clin Invest 1994; 94:2255-2264.

23. Hasselgren P.O., James J.H., Benson D.W., Hall-Angeras M., Angeras U., Hiyama D.T., Li S., Fischer J.E. Total and myofibrillar protein breakdown in different types of skeletal muscle: effects of sepsis and regulation by insulin. Metabolism 1989; 38:634-641.

24. Tiao G, Fagan JM, Samuels N, James JH, Hudson K, Fischer JE, Fischer, Hasselgren PO. Sepsis stimulates non-lysosomal, energy dependent proteolysis and increases ubiquitin mRNA levels in rat skeletal muscle. J Trauma 94, 2255-2264. 1994.

25. Fang CH, Tiao G, James JH, Ogle CK, Fischer JE, Hasselgren PO. Burn injury stimulates multiple proteolytic pathways in skeletal muscle including the ubiquitin-energy-dependent pathway. J Am Coll Surg 180, 161-168. 1995.

26. Mansoor O, Beaufrere B, Boirie Y, Ralliere C, Taillandiere D, Aurousseau E, Schoeffler P, Aarsland AA, Attaix D. Increased mRNA levels for components of the lysosomal, Ca2+ activated, and ATP-ubiquitin-dependent proteolytic pathways in skeletal muscle form head trauma patients. Proc Natl Acad Sci USA 93, 2714-2718. 1996.

27. Gore DC, Honeycutt D, Jahoor F, Wolfe RR, Herndon DN. Effect of exogenous growth hormone on whole-body and isolated-limb protein kinetics in burned patients. Arch Surg 126, 38-43. 1991.

28. Knox J, Demling RH, Wilmore DW, Sarraf P, Santos A. Increased survival after major thermal injury: the effect of growth hormone therapy in adults. J Trauma 39, 526-532. 1995.

29. Demling RH. Comparison of the anabolic effects and complications of human growth hormone and the testosterone analog, oxandrolone, after severe burn injury. Burns 25, 215-221. 1999.

30. Cioffi WG, Gore DC, Pruitt BA, Carrougher G, Guler HP, McManus WF. Insulin-like growth factor-1 lowers protein oxidation in patients with thermal injury. Ann Surg , 310-319. 1994.

31. Debroy M.A., Wolf S.E., Xhang X.J., Chinkes D.L., Ferrando A.A., Wolfe R.R., Herndon D.N. Anabolic effects of insulin-like growth factor in combination with insulin-like growth factor binding protein-3 in severely burned adults. J Trauma 1999

32. Herndon D.N., Ramzy P.I., Debroy M.A., Zheng M., Ferrando A.A., Chinkes D.L., Barret J.P., Wolfe R.R., Wolf S.E. Muscle protein catabolism after severe burn: effects of IGF-1/IGFBP-3 treatment. Ann Surg 1999; 229:713-722.

33. Ferrando A.A., Chinkes D.L., Wolf S.E., Matin S., Herndon D.N., Wolfe R.R. A submaximal dose of insulin promotes net skeletal muscle protein synthesis in patients with severe burns. Ann Surg 1999; 229:11-18.

34. Demling R.H. Comparison of the anabolic effects and complications of human growth hormone and the testosterone analog, oxandrolone, after severe burn injury. Burns 1999; 25:215-221.

35. Knox J., Demling R., Wilmore D.W., Sarraf P., Santos A. Increased survival after major thermal injury: the effect of growth hormone therapy in adults. J Trauma 1995; 39:526-532.

36. Demling RH, Desanti L. Oxandrolone, an anabolic steroid, significantly increases the rate of weight gain in the recovery phase after major burns. J Trauma 43, 47-51. 1997.

37. Balagopal P., Rooyackers O.E., Adey D.B., Ades P.A., Nair K.S. Effects of aging on in vivo synthesis of skeletal muscle myosin heavy chain and sarcoplasmic protein in humans. Am J Physiol 1997; 273:E790-E800.

38. Fang C.H., Li B.G., Wang J.J., Fischer J.E., Hasselgren P.O. Treatment of burned rats with insulin-like growth factor I inhibits the catabolic response in skeletal muscle. Am J Physiol 1998; 275:R1091-R1098.

39. Herndon D.N., Barrow R.E., Kunkel K.R., Broemeling L.D., Rutan R.L. Effects of recombinant human growth hormone on donor site healing in severely burned children. Ann Surg 1990; 212:424-431.

40. Gilpin D.A., Barrow R.E., Rutan R.L., Broemeling L.D., Herndon D.N. Recombinant human growth hormone accelerates wound healing in children with large cutaneous burns. Ann Surg 1994; 223.

41. Pierre EJ, Barrow RE, Hawkins HK, Nguyen TT, Sakurai Y, Desai M, Wolfe RR, Herndon DN. Effects of insulin on wound healing. J Trauma 1998; 44:342-345.

42. Zhang XJ, Sakurai Y, Wolfe RR. An animal model for measurement of protein metabolism in the skin. Surgery 1996; 119:326-332.

43. Zhang XJ, Chinkes DL, Wolf SE, Wolfe RR. Insulin but not growth hormone stimulates protein anabolism in skin wound and muscle. Am J Physiol 276[39], E712-E720. 1999.

44. Deitch E.A. A policy of early excision and grafting in elderly burn patients shortens the hospital stay and improves survival. Burns Incl Therm Inj 1985; 12:109-114.

1. Herndon D.N., Parks D.H. Comparison of serial debridement and autografting and early massive excision with cadaver skin overlay in the treatment of large burns in children. J Trauma 1986; 26:149-152.
2. Klein GL, Herndon DN, Goodman WG, Langman CB, Phillips WA, Dickson IR, Eastell R, Naylor KE, Maloney NA, Desai MH, Benjamin D, Alfrey AC. Histomorphometric and biochemical characterization of bone following acute severe burns in children. Bone 17: 455-460, 1995.
3. Blimkie CJR. Resistance training during preadolescence. Sports Med 15(6): 389-407, 1993.
4. Blimkie CJR. Resistance training during pre-early puberty: efficacy trainability, mechanisms, and persistence. Can J Spt Sci 17(4): 264-279, 1992.
5. Ramsay JA, Blimkie CJR, Smith K, Garner S, MacDougall JD, Sale DG. Strength training effects in prepubescent boys. Med Sci Sports Exerc 22: 605-614, 1990.
6. Maas HCM, De Vries WR, Maitimu I, Bol E, Bowers CY, Koppeschaar HPF. Growth hormone responses during strenuous exercise: The role of growth hormone releasing hormone and growth hormone releasing peptide-z. Med Sci Sports Exerc 2000; 32(7):1226-1232.
7. Atha J. Strengthening muscle. Exer Sports Sci Rev. 9: 1-73, 1981.
8. Chesley A, MacDougall JD, Tarnopolsky MA, Atkinson SA, Smith K. Changes in human muscle protein synthesis after resistance exercise. J Appl Physiol 73: 1383-1388, 1992.
9. Biolo G, Tipton KD, Klein S, Wolfe RR. Enhanced protein synthesis in skeletal muscle after resistance exercise during hyperaminoacidemia: role of blood flow and amino acid transport. Am J Physiol (Endocrinol Metab) 273 (36): E122-E129, 1997.
10. Sale DG. Neural adaptation to strength training. In: Strength and Power in Sports. The Encyclopedia of Sports Medicine. P Komi (ed). Oxford, England: Blackwell, 1992, pp. 249-265.
11. Ferrando AA, Tipton KD, Bamman MM, Wolfe RR. Resistance exercise maintains skeletal muscle protein synthesis during bed rest. J Appl Physiol 82: 807-810, 1997.
12. Phillips SM, Tipton KD, Aarsland AA, Cortiella JC, Wolf SE, Wolfe RR. Mixed muscle protein synthesis and breakdown after resistance exercise in humans. Am J Physiol 273: E99-E107, 1997.
13. Engrav LH, Covey MH, Dutcher KD, Heimbach DM, Walkinshaw MD, Marvin JA. Impairment, time out of school, and time off from work after burns. Plast Recon Surg. 79: 927-932, 1987.
14. Xiao J, Cai BR. Functional and occupational outcome in patients surviving massive burns. Burns 21: 415-421, 1995.
15. Doctor, ME. Returning to school after a severe burn. In, Boswick JA Jr., (Ed.) The Art and Science of Burn Care, Rockville, MD, Aspen Press, 1987: 323-8.
16. Walls Rosenstein DL. A school re-entry program for burned children. Part 1: development and implementation of a school re-entry program. J Burn Care Rehab 8: 319-22, 1987.
17. Fletchall S, Hickerson W. Quality burn rehabilitation: cost effective approach. J Burn Care Rehab 16: 539-542, 1995.
18. Zeller J, Sturm G, Cruse CW. Patients with burns are successful in work hardening programs. J Burn Care Rehab 14:189-196, 1993.
19. Wrigley M, Trotman BK, Dimick A, Fine PR. Factors relating to return to work after burn injury. 16:445-450, 1995.

64. Cheng S, Rogers JC. Changes in occupational role performance after a severe burn: a retrospective study. Am J Occup Ther 43: 17-24, 1989.65.Hart D.W., Wolf S.E., Chinkes D.L., Gore D.C., Desai M.H., Obeng M.K., Lal S.O., Gold W., Ferrando A.A., Wolfe R.R., Herndon D.N. Determinants of catabolism after severe burn. Ann Surg 2000.

66. Carvahal H.F. A physiologic approach to fluid therapy in severely burned children. Surg Gynecol Obstet 1980; 150:379-384.

48. Takala J, Ruokonen E, Webster NR, Nielsen MS, Zandstra DF, Vundelinckx, Hinds CJ. Increased mortality associated with growth hormone treatment in critically ill adults. N Engl J Med 341[11], 785-792. 1999. Ref Type: Generic

49. Ramirez R.J., Wolf S.E., Barrow R.E., Herndon D.N. Growth hormone therapy in burns: a safe therapeutic approach. Ann Surg 1998; 228:439-448.

50. Herndon D.N., Barrow R.E., Stein M., Linares H., Rutan T.C., Rutan R.L., Abston S. Increased mortality with intravenous supplemental feeding in severely burned patients. J Burn Care Rehabil 1989; 10:309-313.

51. Herndon D.N., Stein M., Rutan T.C. Failure of TPN supplementation to improve liver function, immunity, and mortality in thermally injured patients. J Trauma 1987; 27:195-198.

52. Wolf S.E., Jeschke M.G., Rose J.K., Desai M.H., Herndon D.N. Enteral feeding intolerance: An indicator of sepsis associated mortality in burned children. Arch Surg 1997; 132:1310-1314.

53. Haarbo J, Gotfredsen A, Hassager C, Christiansen C. Validation of body composition by dual energy X-ray absorptiometry (DEXA). Clin Physiol 11[4], 331-341. 1991.

54. Ferrando AA, Lane HW, Stuart CA, Wolfe RR. Prolonged bed rest decreases skeletal muscle and whole-body protein synthesis. Am J Physiol 270, E654-E662. 1996.

55. Moore F.D., Oleson K.H., McMurrey J.D. The body cell mass and its supporting environment. Philadelphia: Saunders 1963;22-42.

56. Calder AG, Anderson SE, Grant I, McNurlan MA, Garlick PJ. The determination of low d_5_ -phenylalanine enrichment (0.002-0.09 atom percent excess), after conversion to phenylethylamine, in relation to protein turnover studies by gas chromatography/electron ionization mass spectrometry. Rapid Communications in Mass Spectrometry 6, 421-424. 1992.
